# Supplementary material for: A single-chain and fast-responding light-inducible Cre recombinase as a novel optogenetic switch
Source: eLife. 2021 Feb 23;10:e61268. doi: 10.7554/eLife.61268 (PMC7997657; doi:10.7554/eLife.61268)
Supplement: Supplementary file 1 [file elife-61268-supp1.odt]

Supplementary File

A single-chain and fast-responding light-inducible Cre recombinase as a novel optogenetic switch

Hélène Duplus-Bottin1, Martin Spichty1,*, Gérard Triqueneaux1, Christophe Place2, Philippe Emmanuel Mangeot3, Théophile Ohlmann3, Franck Vittoz2 and Gaël Yvert1,#

1) Laboratory of Biology and Modeling of the Cell, Ecole Normale Supérieure de Lyon, CNRS, Université de Lyon, 69007 Lyon, France.

2) Laboratory of Physics, Ecole Normale Supérieure de Lyon, CNRS, Université de Lyon, 69007 Lyon, France.

3) CIRI-Centre International de Recherche en Infectiologie, Université Claude Bernard Lyon 1, Université de Lyon, Inserm, U1111, CNRS, UMR5308, Ecole Normale Supérieure de Lyon, 69007, Lyon, France

*) Current address: Laboratoire d’Innovation Moléculaire et Applications, Site de Mulhouse – IRJBD, 3 bis rue Alfred Werner, 68057 Mulhouse Cedex, France

#) corresponding author.

Table of Content

Table S1: List of plasmids used in this study.............................................................. p2

Table S2: List of strains used in this study................................................................... p4

Table S3: List of DNA oligonucleotides used in this study.................................... p5-6

Supplementary References................................................................................................. p6

Supplementary Text S1: Synthetic nucleotidic sequences.................................... p7-22

Supplementary Text S2: Peptide sequences................................................................ p23-26

Supplementary Table S1: List of plasmids used in this study.

| ID | System | Type | Selection | Description | Reference |
| --- | --- | --- | --- | --- | --- |
| pRS314 | yeast | CEN/ARS | TRP1 | empty vector | Sikorski et al. 1 |
| pSH47 | yeast | CEN/ARS | URA3 | pGal1:Cre | Gueldener et al. 2 |
| pSH63 | yeast | CEN/ARS | TRP1 | pGal1:Cre | Gueldener et al. 2 |
| pHO-poly-HO | yeast | Integrative | none | Vector for integration at HO locus | Voth et al. 3 |
| pIS385 | yeast | Integrative | URA3 | Vector for integration at LYS2 locus | Sadowski et al.4 |
| Addgene 51269 | human | - | - | pCAG-loxPSTOPloxP-ZsGreen reporter of Cre activity | Hermann et al.5 |
| Addgene 55779 | human | - | NeoR | mCherry-Mem | Yost et al.6 |
| pCDNA5/FRT | human | Integrative (FlpIn) |  | Suitable for single-site insertion | Invitrogen |
| YEplac195-YB_E_I | yeast | 2-micron | URA3 | crtE-crtI-crtYB plasmid | Verwaal et al.7 |
| pGY8 | yeast | Integrative | NAT | PMET17-GFP | Ansel et al. 8 |
| pGY262 | yeast | Integrative | KlLEU2 | HO-L:PTEF-loxP-KlLEU2-STOP-loxP-spHIS5:HO-R | This work |
| pGY286 | yeast | CEN/ARS | TRP1 | pGal1:Cre-VVD-N327T | This work |
| pGY339 | yeast | CEN/ARS | TRP1 | pGal1:Cre-VVD | This work |
| pGY372 | yeast | CEN/ARS | TRP1 | pGal1:CreAA | This work |
| pGY407 | yeast | Integrative | KlLEU2 | HO-L:PTEF-loxP-KlLEU2-STOP-loxP-GFP:HO-R | This work |
| pGY408 | yeast | CEN/ARS | TRP1 | pGal1:LOV2chimJaCre | This work |
| pGY415 | yeast | CEN/ARS | TRP1 | pGal1:LOV_Cre32 | This work |
| pGY416 | yeast | CEN/ARS | TRP1 | pGal1:LiCre | This work |
| pGY417 | yeast | CEN/ARS | URA3 | pGal1:LOV_Cre32 | This work |
| pGY459 | yeast | CEN/ARS | TRP1 | pGal1:LOV2(QIK)_Cre32 | This work |
| pGY461 | yeast | CEN/ARS | TRP1 | pGal1:LOV2(QIIL)_Cre32 | This work |
| pGY462 | yeast | CEN/ARS | TRP1 | pGal1:LOV2(QIVV)_Cre32 | This work |
| pGY463 | yeast | CEN/ARS | TRP1 | pGal1:LOV2(QIAI)_Cre32 | This work |
| pGY464 | yeast | CEN/ARS | TRP1 | pGal1:LOV2(QIQPG)_Cre32 | This work |
| pGY465 | yeast | CEN/ARS | TRP1 | pMet17:LOV_Cre32 | This work |
| pGY466 | yeast | CEN/ARS | TRP1 | pMet17:LiCre | This work |
| pGY472 | yeast | Integrative | KlLEU2 | HO-L:PTEF-loxP-KlLEU2-STOP-loxP-mCherry:HO-R | This work |
| pGY488 | yeast | CEN/ARS | TRP1 | pMet17:Cre18-59-nMag-NLS-T2A-NLS-pMag-Cre60to343 | This work |
| pGY491 | yeast | CEN/ARS | TRP1 | pMet17:NLS-pMag-CreC60-343. | This work |
| pGY501 | yeast | CEN/ARS | URA3 | pMet17: CreN18-59-nMag-NLS | This work |
| pGY502 | yeast | CEN/ARS | TRP1 | pMet17:Cre | This work |
| pGY519 | human | - | - | pCDNA5 FRT loxP5 | This work |
| pGY520 | human | - | - | pCAG-Lox-STOP-MEM cherry-RV | This work |
| pGY521 | human | - | - | pCAG-Lox-STOP-MEM cherry-R1 | This work |
| pGY523 | human | - | - | pCDNA5-FRT-LoxP-1xSTOP-LoxP-MEM cherry RV | This work |
| pGY524 | human | - | - | pCDNA5-FRT-LoxP-1xSTOP-LoxP-MEM cherry R1 | This work |
| pGY525 | human | Integrative (FlpIn) | HygR | pcDNA5-FRT-Lox-Stop-Lox-mCherry-Mem | This work |
| pGY531 | yeast | CEN/ARS | TRP1 | pMet17:CRY2L348F-CreN19-104 | This work |
| pGY532 | yeast | CEN/ARS | URA3 | pMet17:CIB1-CreC106-343 | This work |
| pGY537 | yeast | Integrative | URA3, KlLEU2 | pISLys2:PTEF-loxP-KlLEU2-STOP-loxP-GFP | This work |
| pGY547 | yeast | Template | - | Lox-SynERG9-Lox-tHMG1 | This work |
| pGY553 | yeast | 2-micron | URA3 | pGuid-ERG9-Cas9 | This work |
| pGY559 | yeast | 2-micron | URA3 | YEplac195-PTDH3-loxP-KlLEU2-STOP-loxP-crtYB | This work |
| pGY561 | human | - | G-418 | pCDNA3.1-pCMV-NLSLiCre | This work |
| pGY577 | human | - | - | Lentiviral vector pCMV-NLS-LiCre | This work |
| pGY625 | human | - | - | Lentiviral vector pCMV-MagCre (PA-Cre) | This work |
| pGY626 | human | - | - | Lentiviral vector pCMV-Magnets-opti | This work |

Supplementary Table S2: List of strains used in this study.

| ID | Background | Genotype(i) | Source |
| --- | --- | --- | --- |
| BY4713 | S288c (BY) | MATb leu2∆0 | Brachmann et al. 9 |
| BY4719 | S288c (BY) | MATa trp1∆63 ura3∆0 | Brachmann et al. 9 |
| BY4725 | S288c (BY) | MATa ade2∆::hisG ura3∆0 | Brachmann et al. 9 |
| BY4726 | S288c (BY) | MATb ade2∆::hisG ura3∆0 | Brachmann et al. 9 |
| FYC2-6A | S288c (BY) | MATb his3∆200 leu2∆1 trp1∆63 | Gift from M.T. Teixeira |
| FYC2-6B | S288c (BY) | MATa his3∆200 leu2∆1 trp1∆63 | Gift from M.T. Teixeira |
| GY855 | S288c (BY) | MATa leu2∆0 trp1∆63 ura3∆0 | spore from BY4713 x BY4719 |
| GY983 | S288c (BY) | MATb ade2∆::hisG his3∆200 leu2∆1 trp1∆63 ura3∆0 | spore from BY4725 x FYC2-6A |
| GY984 | S288c (BY) | MATa ade2∆::hisG his3∆200 leu2∆1 trp1∆63 ura3∆0 | spore from BY4726 x FYC2-6B |
| GY1752 | S288c (BY) | MATa ade2∆::hisG his3∆200 leu2∆1 trp1∆63 ura3∆0 ho∆::loxKlLEU2loxGFP | This work |
| GY1761 | S288c (BY) | MATb his3∆200 leu2∆1 trp1∆63 ura3∆0 ho∆::loxKlLEU2loxGFP | GY1752 x FYC2-6A |
| GY2033 | S288c (BY) | MATa his3∆200 leu2∆1 trp1∆63 ho∆::loxKlLEU2loxmCherry | This work |
| GY2206 | S288c (BY) | MATa leu2∆0 lys2∆::loxKlLEU2loxGFP trp1∆63 ura3∆0 | This work |
| GY2207 | S288c (BY) | MATb ade2∆::hisG his3∆200 leu2∆1 trp1∆63 ura3∆0 ho∆::loxKlLEU2loxmCherry | This work |
| GY2214 | S288c (BY) | MATa/MATb ADE2/ade2∆::hisG his3∆200/HIS3 leu2∆0/leu2∆1 LYS2/lys2∆::loxKlLEU2loxGFP trp1∆63/trp1∆63 ura3∆0/ura3∆0 HO/ho∆::loxKlLEU2loxmCherry | GY2206 x GY2207 |
| GY2226 | CEN.PK | MATb his3∆1 leu2-3,112 trp1-289 ura3-52 | Spore from EUROSCARF strain  30000D |
| GY2236 | CEN.PK | MATa his3∆1 leu2-3,112 trp1-289 erg9∆::lox-synERG9-lox-tHMG1 ura3-52::URA3-crtYB-crtI-crtE*[YIplac211-YB/I/E*] | This work |
| GY2247 | CEN.PK | MATa leu2-3,112 trp1-289 ura3-52::URA3-lox-KlLEU2-lox-crtYB-crtI-crtE* | This work |
| Y41388 | CEN.PK | MATa leu2-3,112 trp1-289 ura3-52::URA3-crtYB-crtI-crtE* | EUROSCARF, from Verwaal et al.7 |

(i): MATb corresponds to MATα (alpha)

Supplementary Table S3: List of DNA primers used in this study.

| ID | Sequence(i) (5'-3') |
| --- | --- |
| 1B12 | AAATGTCTTGTCTTCTCTGCTC |
| 1C22 | ACTGTTGCGCGAAGTAGT |
| 1F14 | gtgatgacggtgaaaacctc |
| 1G42 | AGAAAAGGAGAGGGCCAAGA |
| 1J47 | CATGAACTATATCCGTAaCCTGGATAGTGAAACAGGGGC |
| 1J48 | GCCCCTGTTTCACTATCCAGGtTACGGATATAGTTCATG |
| 1J49 | CATGAACTATATCCGTAaCCTGGATtgaGAAACAGGGGCAATGGTGCGCCTGC |
| 1J50 | GCAGGCGCACCATTGCCCCTGTTTCtcaATCCAGGtTACGGATATAGTTCATG |
| 1L71 | gaacatgtccatcaggttcttgcgaacctc-catttaacactcagataa |
| 1L72 | agaaaacgcctggcgatccctgaacatgtc-catttaacactcagataa |
| 1L73 | ggacagaagcattttccaggtatgctcaga-catttaacactcagataa |
| 1L80 | tggtatctttatagtcctgtcgg |
| 1M42 | CTT ATT AAG AAA ACC GCA TTT CAA ATC gcc gat gca acg agt gat gag gtt |
| 1M43 | CTT ATT AAG AAA ACC GCA TTT CAA ATC gcc acg agt gat gag gtt cgc aag |
| 1M44 | CTT ATT AAG AAA ACC GCA TTT CAA ATC gcc agt gat gag gtt cgc aag aac |
| 1M45 | CTT ATT AAG AAA ACC GCA TTT CAA ATC gcc gat gag gtt cgc aag aac ctg |
| 1M46 | CTT ATT AAG AAA ACC GCA TTT CAA ATC gcc gag gtt cgc aag aac ctg atg |
| 1M47 | CTT ATT AAG AAA ACC GCA TTT CAA ATC gcc gtt cgc aag aac ctg atg gac |
| 1M48 | CTT ATT AAG AAA ACC GCA TTT CAA ATC gcc cgc aag aac ctg atg gac atg |
| 1M49 | CTT ATT AAG AAA ACC GCA TTT CAA ATC gcc aag aac ctg atg gac atg ttc |
| 1M50 | CTT ATT AAG AAA ACC GCA TTT CAA ATC gcc aac ctg atg gac atg ttc agg |
| 1M51 | CTT ATT AAG AAA ACC GCA TTT CAA ATC gcc ctg atg gac atg ttc agg gat |
| 1M52 | CTT ATT AAG AAA ACC GCA TTT CAA ATC gcc gac atg ttc agg gat cgc cag |
| 1M53 | CTT ATT AAG AAA ACC GCA TTT CAA ATC gcc agg gat cgc cag gcg ttt tct g |
| 1N24 | CTT ATT AAG AAA ACC GCA TTT CAA ATC VNN AGG GAT CGC CAG GCG TTT TCT G |
| 1N25 | CTT ATT AAG AAA ACC GCA TTT CAA ATC VNN VNN AGG GAT CGC CAG GCG TTT TCT G |
| 1N26 | CTT ATT AAG AAA ACC GCA TTT CAA ATC VNN VNN VNN AGG GAT CGC CAG GCG TTT TCT G |
| 1N95 | acgccaagcgcgcaattaaccctcactaaagggaacaaaagctg-gagctc-cggatgcaagggtt |
| 1N96 | AGAGTTGTTGCTAAAGAACCGTGATGGTGGTGATGATGTGAACCTCTCAT-gtcgaggtcgacggtatcga |
| 1O77 | attaaaagatacgaggcgc |
| 1O80 | atgtgagttacctcactcat |
| 1O82 | CCAGGAGCATACAGTGTGTGACCACCGACTTTTCTCTTCTTCTTTGGTAC-CATGTCGAGGTCGACGGTATC |
| 1O83 | tcgaccggtaatgcaggcaaattttggtgtacggtcagtaaattggacat-gtcgaggtcgacggtatcga |
| 1O89 | aaggatttcaacatcgacg |
| 1P57 | CTGAGGTTCTTCTTTCATATAC |
| 1P58 | CCAGTGAATAATTCTTCACC |
| 1P74 | gttagtcttttttttagttttaaaacaccagaacttagtttcgacggattctag-TTCTCACATCACATCCGAAC |
| 1P75 | agagtatagatcagatggatctggtaatatgcgagagccgtcatggatcc-TTCTTCACCTTTAGAgATGG |
| 1Q74 | TACGGCTGAGTGAAGGCAGTAAG |
| 1Q75 | CTCCTCACGCCGTCTGGTA |
| Sauci | gatatcaccggtaccatgGGTCCAAAAAAGAAGAGAAAGGTAGATCC |
| Flard | gatatcaagcTTAGTCGCCGGCGGCCAGCAGCCTCACCatg |

(i) N = any nucleotide, V = A, C or G.

SUPPLEMENTARY REFERENCES

1.	Sikorski, R. S. & Hieter, P. A system of shuttle vectors and yeast host strains designed for efficient manipulation of DNA in Saccharomyces cerevisiae. Genetics 122, 19–27 (1989).

2.	Gueldener, U., Heinisch, J., Koehler, G. J., Voss, D. & Hegemann, J. H. A second set of loxP marker cassettes for Cre-mediated multiple gene knockouts in budding yeast. Nucleic Acids Res 30, e23 (2002).

3.	Voth, W. P., Richards, J. D., Shaw, J. M. & Stillman, D. J. Yeast vectors for integration at the HO locus. Nucleic Acids Res 29, E59-9 (2001).

4.	Sadowski, I., Su, T. C. & Parent, J. Disintegrator vectors for single-copy yeast chromosomal integration. Yeast 24, 447–55 (2007).

5.	Hermann, M. et al. Binary recombinase systems for high-resolution conditional mutagenesis. Nucleic Acids Res. 42, 3894–3907 (2014).

6.	Yost, E. A., Mervine, S. M., Sabo, J. L., Hynes, T. R. & Berlot, C. H. Live Cell Analysis of G Protein β5 Complex Formation, Function, and Targeting. Mol. Pharmacol. 72, 812–825 (2007).

7.	Verwaal, R. et al. High-Level Production of Beta-Carotene in Saccharomyces cerevisiae by Successive Transformation with Carotenogenic Genes from Xanthophyllomyces dendrorhous. Appl. Environ. Microbiol. 73, 4342–4350 (2007).

8.	Ansel, J. et al. Cell-to-cell stochastic variation in gene expression is a complex genetic trait. PLoS Genet 4, e1000049 (2008).

9.	Brachmann, C. B. et al. Designer deletion strains derived from Saccharomyces cerevisiae S288C: a useful set of strains and plasmids for PCR-mediated gene disruption and other applications. Yeast 14, 115–32 (1998).

Supplementary Text S1: Synthetic nucleotidic sequences cited in Methods.

>CreCVII
GATATCCAAGCTGGTGGCTGGACCAATGTAAATATTGTCATGAACTATATCCGTACCCTGGATAGTGAAACAGGGGCAATGGTGCGCCTGCTGGAAGATGGCGATGGTGGTTCTGGAATGTCTCATACTGTTAATTCTTCTACTATGAATCCATGGGAAGTTGAAGCTTATCAACAATATCATTATGATCCAAGAACTGCTCCAACTGCTAATCCATTGTTCTTTCATACTTTGTATGCTCCAGGAGGTTATGATATTATGGGTTACCTAATTCAAATTATGAATAGACCAAATCCACAAGTGGAATTAGGTCCCGTTGACACCTCCTGCGCCCTAATTTTATGTGACTTGAAGCAAAAAGACACTCCAATTGTGTACGCTAGTGAAGCGTTCCTATACATGACGGGTTATTCTAATGCAGAGGTTCTGGGAAGGAATTGCAGGTTTTTACAGTCTCCTGACGGTATGGTGAAACCAAAATCCACAAGAAAATACGTAGATTCGAACACTATCAACACCATTAGGAAGGCTATCGATAGAAATGCGGAAGTACAGGTTGAAGTCGTAAATTTCAAAAAGAACGGGCAAAGATTTGTAAACTTCTTGACCATTATTCCAGTGAGAGATGAAACAGGTGAATATAGATACTCTATGGGTTTTCAATGTGAAACTGAAGATTATAAAGATGATGATGACAAATAACTCGAGTCATGTAATTAGTTATGTCACGCTTACATTCACGATATC

> CreN-nMag-NLS-T2A-NLS-pMag-CreCpartly AGATCTCAGATACATAGATACAATTCTATTACCCCCATCCATACTCTAGAACTAGTGGATCCCCCGGGCTGCAGGAATTCGATATCAAGCTTATCGATACCGTCGACCTCGACATGGCAACGAGTGATGAGGTTCGCAAGAACCTGATGGACATGTTCAGGGATCGCCAGGCGTTTTCTGAGCATACCTGGAAAATGCTTCTGTCCGTTTGCCGGTCGTGGGCGGCATGGTGCAAGTTGAATGGGACACATACTTTGTACGCTCCAGGGGGCTATGACATTATGGGTTACCTGGATCAAATTGGAAATAGGCCAAACCCTCAGGTGGAACTCGGTCCAGTAGATACTTCATGCGCTTTAATTCTTTGTGACTTAAAACAGAAAGATACACCTATTGTATATGCTTCCGAAGCGTTTTTGTACATGACTGGTTATTCGAATGCTGAAGTTCTAGGTAGGAATTGCAGATTCCTACAATCCCCTGACGGCATGGTGAAGCCTAAATCCACCCGAAAGTACGTCGACTCCAATACCATTAATACGATGAGAAAAGCTATTGATAGAAACGCTGAAGTACAAGTTGAAGTCGTCAATTTTAAGAAGAATGGCCAGAGATTCGTTAACTTCTTGACCATGATTCCAGTAAGAGATGAAACGGGCGAATACAGGTATTCGATGGGTTTTCAATGCGAGACTGAAGGTGGCTCCGGAGGTGTTCCGAAGAAGAAAAGAAAAGTGGGTTCCGGAGAAGGCCGCGGTTCTCTTCTTACTTGTGGAGATGTTGAGGAAAATCCTGGTCCACTCGAGGTACCAAAGAAGAAGAGAAAAGTCGGTGGTCACACACTGTATGCTCCTGGTGGCTATGATATCATGGGTTACCTAAGACAAATTAGAAATCGCCCTAATCCACAAGTCGAATTGGGTCCCGTTGATACCTCTTGCGCTCTTATTTTGTGCGACCTAAAGCAAAAAGACACTCCTATCGTATATGCTTCGGAAGCTTTCCTGTATATGACAGGTTACAGTAACGCCGAAGTTTTGGGGCGAAATTGCAGGTTTCTGCAAAGCCCAGACGGGATGGTGAAGCCCAAAAGTACAAGAAAGTACGTGGACAGCAACACGATAAATACCATGAGAAAAGCCATTGATAGGAATGCCGAAGTACAAGTGGAAGTTGTAAACTTTAAGAAGAATGGACAGAGATTTGTCAATTTCCTAACTATGATCCCGGTTAGGGATGAGACAGGTGAATACCGCTACTCGATGGGATTTCAGTGTGAAACAGAAGGCACGAACCGGAAATGGTTTCCCGCAGAACCTGAAGATGTTCGCGATTATCTTCTATATCTTCAGGCGCGCGGTCTGGCAGTAAAAACTATCCAGCAACATTTGGGCCAGCTAAACATGCTTCATCGTCGGTCCGGGCTGCCACGACCAAGTGACAGCAATGCTGTTTCACTGGTTATGCGGCGGATCCGAAAAGAAAACGTTGATGCCGGTGAACGTGCAAAACAGGCTCTAAGATCT

> EcoRI-LovCre_chimJa-BstBI

GAATTCGATATCAAGCTTATCGATACCGTCGAGGGGCAGAGCCGATCCTGTACACTTTACTTAAAACCATTATCTGAGTGTTAAATGAGAGGTTCACATCATCACCACCATCACGGTTCTTTAGCAACAACTCTGGAAAGAATCGAGAAAAACTTCGTCATCACGGACCCGAGGCTACCCGACAACCCTATCATTTTCGCATCCGACTCTTTTTTACAATTAACTGAATATTCTAGAGAAGAAATTCTGGGCCGTAATTGTAGGTTTTTACAGGGTCCAGAAACAGATAGAGCAACAGTTAGAAAGATTAGAGATGCTATTGATAATCAGACTGAGGTAACAGTTCAACTAATAAACTATACTAAATCCGGGAAGAAATTTTGGAATGTCTTCCACTTACAGCCAATGAGAGATTACAAAGGTGATGTCCAGTATTTTATCGGAGTTCAGTTAGACGGCACAGAACGTCTGCACGGTGCCGCGGAGAGGGAAGCTGTTTGTCTTATTAAGAAAACCGCATTTCAAATCGACGAAGCCGTACGTGACAGACAAGCATTTTCTGAGCATACCTGGAAAATGCTTCTGTCCGTTTGCCGGTCGTGGGCGGCATGGTGCAAGTTGAATAACCGGAAATGGTTTCCCGCAGAACCTGAAGATGTTCGCGATTATCTTCTATATCTTCAGGCGCGCGGTCTGGCAGTAAAAACTATCCAGCAACATTTGGGCCAGCTAAACATGCTTCATCGTCGGTCCGGGCTGCCACGACCAAGTGACAGCAATGCTGTTTCACTGGTTATGCGGCGGATCCGAAAAGAAAACGTTGATGCCGGTGAACGTGCAAAACAGGCTCTAGCGTTCGAA

>LoxLEULoxHIS

GGATCCGACATGGAGGCCCAGAATACCCTCCTTGACAGTCTTGACGTGCGCAGCTCAGGGGCATGATGTGACTGTCGCCCGTACATTTAGCCCATACATCCCCATGTATAATCATTTGCATCCATACATTTTGATGGCCGCACGGCGCGAAGCAAAAATTACGGCTCCTCGCTGCAGACCTGCGAGCAGGGAAACGCTCCCCTCACAGACGCGTTGAATTGTCCCCACGCCGCGCCCCTGTAGAGAAATATAAAAGGTTAGGATTTGCCACTGAGGTTCTTCTTTCATATACTTCCTTTTAAAATCTTGCTAGGATACAGTTCTCACATCACATCCGAACATAAACAACCGTTAACATAACTTCGTATAGCATACATTATACGAAGTTATCCATGTCTAAGAATATCGTTGTCCTACCGGGTGATCACGTCGGTAAAGAAGTTACTGACGAAGCTATTAAGGTCTTGAATGCCATTGCTGAAGTCCGTCCAGAAATTAAGTTCAATTTCCAACATCACTTGATCGGGGGTGCTGCCATCGATGCCACTGGCACTCCTTTACCAGATGAAGCTCTAGAAGCCTCTAAGAAAGCCGATGCTGTCTTACTAGGTGCTGTTGGTGGTCCAAAATGGGGTACGGGCGCAGTTAGACCAGAACAAGGTCTATTGAAGATCAGAAAGGAATTGGGTCTATACGCCAACTTGAGACCATGTAACTTTGCTTCTGATTCTTTACTAGATCTTTCTCCTTTGAAGCCTGAATATGCAAAGGGTACCGATTTCGTCGTCGTTAGAGAATTGGTTGGTGGTATCTACTTTGGTGAAAGAAAAGAAGATGAAGGTGACGGAGTTGCTTGGGACTCTGAGAAATACAGTGTTCCTGAAGTTCAAAGAATTACAAGAATGGCTGCTTTCTTGGCATTGCAACAAAACCCACCATTACCAATCTGGTCTCTTGACAAGGCTAACGTGCTTGCCTCTTCCAGATTGTGGAGAAAGACTGTTGAAGAAACCATCAAGACTGAGTTCCCACAATTAACTGTTCAGCACCAATTGATCGACTCTGCTGCTATGATTTTGGTTAAATCACCAACTAAGCTAAACGGTGTTGTTATTACCAACAACATGTTTGGTGATATTATCTCCGATGAAGCCTCTGTTATTCCAGGTTCTTTGGGTTTATTACCTTCTGCATCTCTAGCTTCCCTACCTGACACTAACAAGGCATTCGGTTTGTACGAACCATGTCATGGTTCTGCCCCAGATTTACCAGCAAACAAGGTTAATCCAATTGCTACCATCTTATCTGCAGCTATGATGTTGAAGTTATCCTTGGATTTGGTTGAAGAAGGTAGGGCTCTTGAAGAAGCTGTTAGAAATGTCTTGGATGCAGGTGTCAGAACCGGTGACCTTGGTGGTTCTAACTCTACCACTGAGGTTGGCGATGCTATCGCCAAGGCTGTCAAGGAAATCTTGGCTTAACGCGCCACTTCTAAATAAGCGAATTTCTTATGATTTATGATTTTTATTATTAAATAAGTTATAAAAAAAATAAGTGTATACAAATTTTAAAGTGACTCTTAGGTTTTAAAACGAAAATTCTTGTTCTTGAGTAACTCTTTCCTGTAGGTCAGGTTGCTTTCTCAGGTATAGCATGAGGTCGCTCTTATTGACCACACCTCTACCGGCAGATCGCTAGCATAACTTCGTATAGCATACATTATACGAAGTTATCCATGGGTAGGAGGGCTTTTGTAGAAAGAAATACGAACGAAACGAAAATCAGCGTTGCCATCGCTTTGGACAAAGCTCCCTTACCTGAAGAGTCGAATTTTATTGATGAACTTATAACTTCCAAGCATGCAAACCAAAAGGGAGAACAAGTAATCCAAGTAGACACGGGAATTGGATTCTTGGATCACATGTATCATGCACTGGCTAAACATGCAGGCTGGAGCTTACGACTTTACTCAAGAGGTGATTTAATCATCGATGATCATCACACTGCAGAAGATACTGCTATTGCACTTGGTATTGCATTCAAGCAGGCTATGGGTAACTTTGCCGGCGTTAAAAGATTTGGACATGCTTATTGTCCACTTGACGAAGCTCTTTCTAGAAGCGTAGTTGACTTGTCGGGACGGCCCTATGCTGTTATCGATTTGGGATTAAAGCGTGAAAAGGTTGGGGAATTGTCCTGTGAAATGATCCCTCACTTACTATATTCCTTTTCGGTAGCAGCTGGAATTACTTTGCATGTTACCTGCTTATATGGTAGTAATGACCATCATCGTGCTGAAAGCGCTTTTAAATCTCTGGCTGTTGCCATGCGCGCGGCTACTAGTCTTACTGGAAGTTCTGAAGTCCCAAGCACGAAGGGAGTGTTGTAATAACTGGTCGAGTCATGTAATTAGTTATGTCACGCTTACATTCACGCCCTCCCCCCACATCCGCTCTAACCGAAAAGGAAGGAGTTAGACAACCTGAAGTCTAGGTCCCTATTTATTTTTTTATAGTTATGTTAGTATTAAGAACGTTATTTATATTTCAAATTTTTCTTTTTTTTCTGTACAGACGCGTGTACGCATGTAACATTATACTGAAAACCTTGCTTGAGAAGGTTTTGGGACGCTCGAAGGCTTTAATTTGCGGCCGGTACGGATCC

>LEULoxGreen

GCTAGCATAACTTCGTATAGCATACATTATACGAAGTTATCCATGTCTAAAGGTGAAGAATTATTCACTGGTGTTGTCCCAATTTTGGTTGAATTAGATGGTGATGTTAATGGTCACAAATTTTCTGTCTCCGGTGAAGGTGAAGGTGATGCTACTTACGGTAAATTGACCTTAAAATTTATTTGTACTACTGGTAAATTGCCAGTTCCATGGCCAACCTTAGTCACTACTTTCGGTTATGGTGTTCAATGTTTTGCTAGATACCCAGATCATATGAAACAACATGACTTTTTCAAGTCTGCCATGCCAGAAGGTTATGTTCAAGAAAGAACTATTTTTTTCAAAGATGACGGTAACTACAAGACCAGAGCTGAAGTCAAGTTTGAAGGTGATACCTTAGTTAATAGAATCGAATTAAAAGGTATTGATTTTAAAGAAGATGGTAACATTTTAGGTCACAAATTGGAATACAACTATAACTCTCACAATGTTTACATCATGGCTGACAAACAAAAGAATGGTATCAAAGTTAACTTCAAAATTAGACACAACATTGAAGATGGTTCTGTTCAATTAGCTGACCATTATCAACAAAATACTCCAATTGGTGATGGTCCAGTCTTGTTACCAGACAACCATTACTTATCCACTCAATCTGCCTTATCCAAAGATCCAAACGAAAAGAGAGACCACATGGTCTTGTTAGAATTTGTTACTGCTGCTGGTATTACCCATGGTATGGATGAATTGTACAAATAACCTAGGCTGGTCGAGTCATGTAATTAGTTATGTCACGCTTACATTCACGCCCTCCCCCCACATCCGCTCTAACCGAAAAGGAAGGAGTTAGACAACCTGAAGTCTAGGTCCCTATTTATTTTTTTATAGTTATGTTAGTATTAAGAACGTTATTTATATTTCAAATTTTTCTTTTTTTTCTGTACAGACGCGTGTACGCATGTAACATTATACTGAAAACCTTGCTTGAGAAGGTTTTGGGACGCTCGAAGGCTTTAATTTGCGGCCGGTACGAGCTC

>LEULoxmCherry

ACCGGTGACCTTGGTGGTTCTAACTCTACCACTGAGGTTGGCGATGCTATCGCCAAGGCTGTCAAGGAAATCTTGGCTTAACGCGCCACTTCTAAATAAGCGAATTTCTTATGATTTATGATTTTTATTATTAAATAAGTTATAAAAAAAATAAGTGTATACAAATTTTAAAGTGACTCTTAGGTTTTAAAACGAAAATTCTTGTTCTTGAGTAACTCTTTCCTGTAGGTCAGGTTGCTTTCTCAGGTATAGCATGAGGTCGCTCTTATTGACCACACCTCTACCGGCAGATCGCTAGCATAACTTCGTATAGCATACATTATACGAAGTTATCCATGGTGTCAAAGGGGGAGGAAGATAATATGGCGATAATTAAAGAGTTCATGAGGTTTAAAGTCCACATGGAGGGTTCAGTCAACGGTCATGAGTTCGAGATCGAAGGTGAGGGTGAAGGCAGACCGTATGAAGGTACTCAAACTGCCAAATTGAAGGTGACCAAAGGCGGCCCACTTCCGTTTGCGTGGGACATTCTTTCACCTCAATTCATGTACGGTTCGAAAGCTTATGTTAAACATCCAGCAGATATTCCCGATTATCTAAAGTTGTCTTTCCCTGAAGGTTTTAAATGGGAAAGGGTCATGAATTTTGAAGACGGTGGGGTTGTAACGGTAACACAGGATTCCTCATTACAAGATGGCGAATTTATCTATAAAGTCAAGTTGCGTGGCACTAATTTTCCTTCTGATGGTCCTGTCATGCAGAAGAAAACAATGGGCTGGGAAGCTTCAAGCGAAAGGATGTACCCAGAAGATGGTGCTTTAAAGGGTGAGATCAAACAAAGATTAAAGTTAAAGGACGGCGGGCATTACGATGCTGAAGTTAAAACGACTTATAAAGCTAAGAAACCTGTTCAGCTGCCAGGTGCATACAACGTGAATATAAAGCTTGACATAACATCACATAACGAGGACTATACAATTGTTGAACAGTATGAAAGAGCCGAAGGTCGTCACAGTACTGGAGGGATGGATGAACTATACAAATAACCTAGGCTGGTCGAGTCATGTAATTAGTTATGTCACGCTTACATTCACGCCCTCCCCCCACATCCGCTCTAACCGAAAAGGAAGGAGTTAGACAACCTGAAGTCTAGGTCCCTATTTATTTTTTTATAGTTATGTTAGTATTAAGAACGTTATTTATATTTCAAATTTTTCTTTTTTTTCTGTACAGACGCGTGTACGCATGTAACATTATACTGAAAACCTTGCTTGAGAAGGTTTTGGGACGCTCGAAGGCTTTAATTTGCGGCCGGTACGAGCTCGAATTC

>CIB1CreCter

TCGCGCGTTTCGGTGATGACGGTGAAAACCTCTGACACATGCAGCTCCCGGAGACGGTCACAGCTTGTCTGTAAGCGGATGCCGGGAGCAGACAAGCCCGTCAGGGCGCGTCAGCGGGTGTTGGCGGGTGTCGGGGCTGGCTTAACTATGCGGCATCAGAGCAGATTGTACTGAGAGTGCACCATATGCGGTGTGAAATACCGCACAGATGCGTAAGGAGAAAATACCGCATCAGGCGCCATTCGCCATTCAGGCTGCGCAACTGTTGGGAAGGGCGATCGGTGCGGGCCTCTTCGCTATTACGCCAGCTGGCGAAAGGGGGATGTGCTGCAAGGCGATTAAGTTGGGTAACGCCAGGGTTTTCCCAGTCACGACGTTGTAAAACGACGGCCAGTAGATCTACGCCAAGCGCGCAATTAACCCTCACTAAAGGGAACAAAAGCTGGAGCTCCGGATGCAAGGGTTCGAATCCCTTAGCTCTCATTATTTTTTGCTTTTTCTCTTGAGGTCACATGATCGCAAAATGGCAAATGGCACGTGAAGCTGTCGATATTGGGGAACTGTGGTGGTTGGCAAATGACTAATTAAGTTAGTCAAGGCGCCATCCTCATGAAAACTGTGTAACATAATAACCGAAGTGTCGAAAAGGTGGCACCTTGTCCAATTGAACACGCTCGATGAAAAAAATAAGATATATATAAGGTTAAGTAAAGCGTCTGTTAGAAAGGAAGTTTTTCCTTTTTCTTGCTCTCTTGTCTTTTCATCTACTATTTCCTTCGTGTAATACAGGGTCGTCAGATACATAGATACAATTCTATTACCCCCATCCATACTCTAGAACTAGTGGATCCCCCGGGCTGCAGGAATTCGATATCAAGCTTATCGATACCGTCGACCTCGACATGAATGGCGCTATTGGCGGTGACCTATTATTAAATTTTCCAGATATGTCTGTCCTAGAAAGACAACGTGCCCATTTAAAATACTTAAATCCTACATTCGATTCGCCACTTGCGGGTTTCTTTGCTGATAGCAGCATGATAACAGGCGGTGAAATGGATAGTTATTTATCCACAGCAGGTCTAAACCTACCCATGATGTATGGGGAAACGACAGTTGAGGGCGATTCTCGTTTGTCAATCTCCCCAGAGACGACCCTTGGCACAGGTAATTTCAAAAAAAGAAAATTTGATACTGAAACCAAGGACTGCAATGAGAAGAAAAAAAAAATGACAATGAATAGGGATGATTTAGTAGAAGAGGGAGAGGAGGAAAAATCCAAAATTACTGAACAAAACAATGGTTCTACCAAGTCGATTAAGAAAATGAAGCATAAGGCAAAAAAAGAAGAGAACAATTTTTCAAATGATTCATCAAAGGTTACTAAAGAATTAGAAAAGACAGATTACATTCACGTTAGAGCACGTAGGGGACAGGCTACTGACTCTCATTCCATTGCGGAGAGAGTCAGGCGTGAAAAAATCTCAGAAAGAATGAAGTTCTTACAGGACCTAGTTCCTGGGTGTGACAAGATTACGGGTAAAGCTGGTATGTTAGACGAGATCATCAACTATGTTCAAAGTCTTCAAAGGCAGATTGAATTCTTATCTATGAAGCTGGCTATCGTAAATCCTAGGCCAGATTTCGATATGGATGATATTTTCGCAAAAGAAGTCGCAAGTACCCCAATGACTGTGGTACCCAGCCCAGAAATGGTGTTGTCTGGTTATTCACACGAAATGGTTCACAGTGGGTACAGCTCAGAGATGGTGAATTCTGGCTACCTTCATGTTAACCCAATGCAACAAGTGAATACGTCCTCTGATCCATTATCCTGTTTTAATAATGGTGAAGCGCCAAGTATGTGGGATTCTCATGTTCAAAATCTATATGGAAATTTGGGAGTAGGTGGTGGGGGTAGCGGAGGCGGTGGTAGCGGTGGTGGTGGAAGGCGACCAAGTGACAGCAATGCTGTTTCACTGGTTATGCGGCGGATCCGAAAAGAAAACGTTGATGCCGGTGAACGTGCAAAACAGGCTCTAGCGTTCAGATCTGGCGTAATCATGGTCATAGCTGTTTCCTGTGTGAAATTGTTATCCGCTCACAATTCCACACAACATACGAGCCGGAAGCATAAAGTGTAAAGCCTGGGGTGCCTAATGAGTGAGCTAACTCACATTAATTGCGTTGCGCTCACTGCCCGCTTTCCAGTCGGGAAACCTGTCGTGCCAGCTGCATTAATGAATCGGCCAACGCGCGGGGAGAGGCGGTTTGCGTATTGGGCGCTCTTCCGCTTCCTCGCTCACTGACTCGCTGCGCTCGGTCGTTCGGCTGCGGCGAGCGGTATCAGCTCACTCAAAGGCGGTAATACGGTTATCCACAGAATCAGGGGATAACGCAGGAAAGAACATGTGAGCAAAAGGCCAGCAAAAGGCCAGGAACCGTAAAAAGGCCGCGTTGCTGGCGTTTTTCCATAGGCTCCGCCCCCCTGACGAGCATCACAAAAATCGACGCTCAAGTCAGAGGTGGCGAAACCCGACAGGACTATAAAGATACCAGGCGTTTCCCCCTGGAAGCTCCCTCGTGCGCTCTCCTGTTCCGACCCTGCCGCTTACCGGATACCTGTCCGCCTTTCTCCCTTCGGGAAGCGTGGCGCTTTCTCATAGCTCACGCTGTAGGTATCTCAGTTCGGTGTAGGTCGTTCGCTCCAAGCTGGGCTGTGTGCACGAACCCCCCGTTCAGCCCGACCGCTGCGCCTTATCCGGTAACTATCGTCTTGAGTCCAACCCGGTAAGACACGACTTATCGCCACTGGCAGCAGCCACTGGTAACAGGATTAGCAGAGCGAGGTATGTAGGCGGTGCTACAGAGTTCTTGAAGTGGTGGCCTAACTACGGCTACACTAGAAGAACAGTATTTGGTATCTGCGCTCTGCTGAAGCCAGTTACCTTCGGAAAAAGAGTTGGTAGCTCTTGATCCGGCAAACAAACCACCGCTGGTAGCGGTGGTTTTTTTGTTTGCAAGCAGCAGATTACGCGCAGAAAAAAAGGATCTCAAGAAGATCCTTTGATCTTTTCTACGGGGTCTGACGCTCAGTGGAACGAAAACTCACGTTAAGGGATTTTGGTCATGAGATTATCAAAAAGGATCTTCACCTAGATCCTTTTAAATTAAAAATGAAGTTTTAAATCAATCTAAAGTATATATGAGTAAACTTGGTCTGACAGTTACCAATGCTTAATCAGTGAGGCACCTATCTCAGCGATCTGTCTATTTCGTTCATCCATAGTTGCCTGACTCCCCGTCGTGTAGATAACTACGATACGGGAGGGCTTACCATCTGGCCCCAGTGCTGCAATGATACCGCGAGACCCACGCTCACCGGCTCCAGATTTATCAGCAATAAACCAGCCAGCCGGAAGGGCCGAGCGCAGAAGTGGTCCTGCAACTTTATCCGCCTCCATCCAGTCTATTAATTGTTGCCGGGAAGCTAGAGTAAGTAGTTCGCCAGTTAATAGTTTGCGCAACGTTGTTGCCATTGCTACAGGCATCGTGGTGTCACGCTCGTCGTTTGGTATGGCTTCATTCAGCTCCGGTTCCCAACGATCAAGGCGAGTTACATGATCCCCCATGTTGTGCAAAAAAGCGGTTAGCTCCTTCGGTCCTCCGATCGTTGTCAGAAGTAAGTTGGCCGCAGTGTTATCACTCATGGTTATGGCAGCACTGCATAATTCTCTTACTGTCATGCCATCCGTAAGATGCTTTTCTGTGACTGGTGAGTACTCAACCAAGTCATTCTGAGAATAGTGTATGCGGCGACCGAGTTGCTCTTGCCCGGCGTCAATACGGGATAATACCGCGCCACATAGCAGAACTTTAAAAGTGCTCATCATTGGAAAACGTTCTTCGGGGCGAAAACTCTCAAGGATCTTACCGCTGTTGAGATCCAGTTCGATGTAACCCACTCGTGCACCCAACTGATCTTCAGCATCTTTTACTTTCACCAGCGTTTCTGGGTGAGCAAAAACAGGAAGGCAAAATGCCGCAAAAAAGGGAATAAGGGCGACACGGAAATGTTGAATACTCATACTCTTCCTTTTTCAATATTATTGAAGCATTTATCAGGGTTATTGTCTCATGAGCGGATACATATTTGAATGTATTTAGAAAAATAAACAAATAGGGGTTCCGCGCACATTTCCCCGAAAAGTGCCACCTGACGTCTAAGAAACCATTATTATCATGACATTAACCTATAAAAATAGGCGTATCACGAGGCCCTTTCGTC

>CRY2CreNter

TCGCGCGTTTCGGTGATGACGGTGAAAACCTCTGACACATGCAGCTCCCGGAGACGGTCACAGCTTGTCTGTAAGCGGATGCCGGGAGCAGACAAGCCCGTCAGGGCGCGTCAGCGGGTGTTGGCGGGTGTCGGGGCTGGCTTAACTATGCGGCATCAGAGCAGATTGTACTGAGAGTGCACCATATGCGGTGTGAAATACCGCACAGATGCGTAAGGAGAAAATACCGCATCAGGCGCCATTCGCCATTCAGGCTGCGCAACTGTTGGGAAGGGCGATCGGTGCGGGCCTCTTCGCTATTACGCCAGCTGGCGAAAGGGGGATGTGCTGCAAGGCGATTAAGTTGGGTAACGCCAGGGTTTTCCCAGTCACGACGTTGTAAAACGACGGCCAGTAGATCTCAGATACATAGATACAATTCTATTACCCCCATCCATACTCTAGAACTAGTGGATCCCCCGGGCTGCAGGAATTCGATATCAAGCTTATCGATACCGTCGACCTCGACATGAAGATGGATAAGAAGACAATCGTTTGGTTCAGAAGGGACTTAAGAATCGAAGACAATCCCGCGTTGGCGGCCGCCGCACATGAGGGATCCGTATTTCCGGTGTTTATCTGGTGCCCAGAAGAAGAAGGGCAATTCTACCCTGGTCGTGCTAGTAGGTGGTGGATGAAGCAATCATTAGCCCACCTCTCCCAATCACTTAAAGCACTGGGCTCTGATCTCACATTGATTAAGACACATAATACGATCTCAGCGATCCTCGACTGCATCAGAGTAACCGGCGCTACAAAGGTTGTGTTCAATCACTTATACGACCCTGTCTCATTGGTCAGAGATCATACCGTAAAAGAGAAACTCGTGGAAAGAGGAATTAGCGTTCAGTCTTATAACGGCGATCTATTGTATGAACCATGGGAAATATACTGTGAAAAAGGGAAGCCATTTACTTCATTTAACTCATATTGGAAAAAATGCCTAGATATGTCTATCGAATCGGTCATGTTGCCTCCCCCCTGGAGGCTAATGCCAATAACCGCAGCTGCTGAAGCTATTTGGGCGTGCAGTATTGAAGAATTGGGGTTAGAAAATGAAGCTGAAAAACCTTCCAATGCATTACTCACAAGAGCTTGGAGTCCCGGATGGTCAAATGCAGATAAATTACTGAATGAATTTATCGAGAAACAATTGATTGATTACGCGAAGAATTCCAAAAAAGTCGTGGGAAACTCTACCAGTTTGCTTTCCCCATATCTCCATTTCGGCGAGATTTCAGTCAGACATGTATTTCAGTGTGCAAGAATGAAACAGATCATTTGGGCAAGGGATAAAAACTCGGAAGGTGAAGAATCTGCTGATCTTTTCTTAAGGGGTATTGGTTTGAGAGAATACTCGCGTTACATCTGCTTCAACTTTCCATTTACGCACGAACAATCATTGTTATCGCATCTGAGGTTTTTCCCATGGGATGCAGATGTCGATAAGTTTAAAGCTTGGAGACAAGGGAGGACAGGTTATCCTCTGGTTGATGCAGGCATGCGTGAATTTTGGGCTACAGGTTGGATGCACAACAGGATAAGGGTTATCGTGTCATCATTCGCCGTAAAGTTTCTTTTACTGCCGTGGAAGTGGGGGATGAAGTACTTTTGGGATACATTGCTTGATGCTGACCTCGAGTGCGACATACTAGGATGGCAGTATATTTCTGGTAGTATTCCAGATGGTCATGAACTTGATCGTTTAGATAATCCCGCTTTGCAGGGTGCCAAATATGATCCAGAAGGTGAATACATTAGGCAGTGGTTACCTGAGTTGGCTCGGCTGCCTACTGAATGGATACACCATCCCTGGGATGCTCCTTTAACTGTCCTGAAAGCATCAGGTGTCGAATTAGGTACCAATTACGCGAAACCTATTGTGGACATCGACACTGCAAGAGAACTCTTGGCCAAAGCGATATCCAGGACACGAGAAGCGCAGATCATGATCGGGGCCGCTCCAGATGAAATCGTTGCCGACTCTTTTGAAGCATTAGGCGCCAATACTATTAAAGAACCAGGGCTATGTCCCAGCGTATCCTCCAATGACCAACAGGTACCATCTGCTGTAAGATATAATGGTTCAAAAAGAGTTAAACCCGAAGAAGAAGAAGAGCGCGACATGAAGAAAAGCAGAGGCTTTGACGAAAGAGAACTATTCTCTACCGCGGAAAGTTCCAGTTCCTCCTCAGTCTTTTTCGTTTCGCAGTCCTGTTCTTTAGCATCTGAAGGTAAGAACCTTGAGGGAATTCAAGATTCTTCTGATCAAATCACCACTTCCCTGGGCAAAAATGGTTGCAAGGGCGGTGGAGGGTCCGGTGGGGGTGGAAGCGGAGGAGGTGGTAGGACGAGTGATGAGGTTCGCAAGAACCTGATGGACATGTTCAGGGATCGCCAGGCGTTTTCTGAGCATACCTGGAAAATGCTTCTGTCCGTTTGCCGGTCGTGGGCGGCATGGTGCAAGTTGAATAACCGGAAATGGTTTCCCGCAGAACCTGAAGATGTTCGCGATTATCTTCTATATCTTCAGGCGCGCGGTCTGGCAGTAAAAACTATCCAGCAACATTTGGGCCAGCTAAACATGCTTCATCGTCGGTCCGGGCTGTAGCCATTAACGCGTAAATGATTGCTATAATTATTTGATATTTATGGTGACATATGAGAAAGGATTTCAACATCGACGGAAAATATGTAGTGCTGTCTGTAAAGATCTGGCGTAATCATGGTCATAGCTGTTTCCTGTGTGAAATTGTTATCCGCTCACAATTCCACACAACATACGAGCCGGAAGCATAAAGTGTAAAGCCTGGGGTGCCTAATGAGTGAGCTAACTCACATTAATTGCGTTGCGCTCACTGCCCGCTTTCCAGTCGGGAAACCTGTCGTGCCAGCTGCATTAATGAATCGGCCAACGCGCGGGGAGAGGCGGTTTGCGTATTGGGCGCTCTTCCGCTTCCTCGCTCACTGACTCGCTGCGCTCGGTCGTTCGGCTGCGGCGAGCGGTATCAGCTCACTCAAAGGCGGTAATACGGTTATCCACAGAATCAGGGGATAACGCAGGAAAGAACATGTGAGCAAAAGGCCAGCAAAAGGCCAGGAACCGTAAAAAGGCCGCGTTGCTGGCGTTTTTCCATAGGCTCCGCCCCCCTGACGAGCATCACAAAAATCGACGCTCAAGTCAGAGGTGGCGAAACCCGACAGGACTATAAAGATACCAGGCGTTTCCCCCTGGAAGCTCCCTCGTGCGCTCTCCTGTTCCGACCCTGCCGCTTACCGGATACCTGTCCGCCTTTCTCCCTTCGGGAAGCGTGGCGCTTTCTCATAGCTCACGCTGTAGGTATCTCAGTTCGGTGTAGGTCGTTCGCTCCAAGCTGGGCTGTGTGCACGAACCCCCCGTTCAGCCCGACCGCTGCGCCTTATCCGGTAACTATCGTCTTGAGTCCAACCCGGTAAGACACGACTTATCGCCACTGGCAGCAGCCACTGGTAACAGGATTAGCAGAGCGAGGTATGTAGGCGGTGCTACAGAGTTCTTGAAGTGGTGGCCTAACTACGGCTACACTAGAAGAACAGTATTTGGTATCTGCGCTCTGCTGAAGCCAGTTACCTTCGGAAAAAGAGTTGGTAGCTCTTGATCCGGCAAACAAACCACCGCTGGTAGCGGTGGTTTTTTTGTTTGCAAGCAGCAGATTACGCGCAGAAAAAAAGGATCTCAAGAAGATCCTTTGATCTTTTCTACGGGGTCTGACGCTCAGTGGAACGAAAACTCACGTTAAGGGATTTTGGTCATGAGATTATCAAAAAGGATCTTCACCTAGATCCTTTTAAATTAAAAATGAAGTTTTAAATCAATCTAAAGTATATATGAGTAAACTTGGTCTGACAGTTACCAATGCTTAATCAGTGAGGCACCTATCTCAGCGATCTGTCTATTTCGTTCATCCATAGTTGCCTGACTCCCCGTCGTGTAGATAACTACGATACGGGAGGGCTTACCATCTGGCCCCAGTGCTGCAATGATACCGCGAGACCCACGCTCACCGGCTCCAGATTTATCAGCAATAAACCAGCCAGCCGGAAGGGCCGAGCGCAGAAGTGGTCCTGCAACTTTATCCGCCTCCATCCAGTCTATTAATTGTTGCCGGGAAGCTAGAGTAAGTAGTTCGCCAGTTAATAGTTTGCGCAACGTTGTTGCCATTGCTACAGGCATCGTGGTGTCACGCTCGTCGTTTGGTATGGCTTCATTCAGCTCCGGTTCCCAACGATCAAGGCGAGTTACATGATCCCCCATGTTGTGCAAAAAAGCGGTTAGCTCCTTCGGTCCTCCGATCGTTGTCAGAAGTAAGTTGGCCGCAGTGTTATCACTCATGGTTATGGCAGCACTGCATAATTCTCTTACTGTCATGCCATCCGTAAGATGCTTTTCTGTGACTGGTGAGTACTCAACCAAGTCATTCTGAGAATAGTGTATGCGGCGACCGAGTTGCTCTTGCCCGGCGTCAATACGGGATAATACCGCGCCACATAGCAGAACTTTAAAAGTGCTCATCATTGGAAAACGTTCTTCGGGGCGAAAACTCTCAAGGATCTTACCGCTGTTGAGATCCAGTTCGATGTAACCCACTCGTGCACCCAACTGATCTTCAGCATCTTTTACTTTCACCAGCGTTTCTGGGTGAGCAAAAACAGGAAGGCAAAATGCCGCAAAAAAGGGAATAAGGGCGACACGGAAATGTTGAATACTCATACTCTTCCTTTTTCAATATTATTGAAGCATTTATCAGGGTTATTGTCTCATGAGCGGATACATATTTGAATGTATTTAGAAAAATAAACAAATAGGGGTTCCGCGCACATTTCCCCGAAAAGTGCCACCTGACGTCTAAGAAACCATTATTATCATGACATTAACCTATAAAAATAGGCGTATCACGAGGCCCTTTCGTC

>gERG9

ggatcctagactctcgaggcgaatttcttatgatttatgatttttattattaaataagttataaaaaaaataagtgtatacaaattttaaagtgactcttaggttttaaaacgaaaattcttattcttgagtaactctttcctgtaggtcaggttgctttctcaggtatagcatgaggtcgctcttattgaccacacctctaccggcatgccgagcaaatgcctgcaaatcgctccccatttctctagagcggccgtggtatcgtttagattggcaattacagACgtcttagctcacatgcttataactaattacatgactcgaagacataaaaaacaaaaaaagcaccaccgactcggtgccactttttcaagttgataacggactagccttattttaacttgctatttCTAGCTCTAAAACATTGTAATAGCTTTCCCATtgatcatttatctttcactgcggagaagtttcgaacgccgaaacatgcgcaccaactttcacttctacagcgtttgaccaaaatcttttgaacagaacattgtagggtgtgaaaaaatgcgcacctttaccgctagc

>pGY577

GGAAATTGTAAACGTTAATATTTTGTTAAAATTCGCGTTAAATTTTTGTTAAATCAGCTCATTTTTTAACCAATAGGCCGAAATCGGCAAAATCCCTTATAAATCAAAAGAATAGACCGAGATAGGGTTGAGTGTTGTTCCAGTTTGGAACAAGAGTCCACTATTAAAGAACGTGGACTCCAACGTCAAAGGGCGAAAAACCGTCTATCAGGGCGATGGCCCACTACGTGAACCATCACCCTAATCAAGTTTTTTGGGGTCGAGGTGCCGTAAAGCACTAAATCGGAACCCTAAAGGGAGCCCCCGATTTAGAGCTTGACGGGGAAAGCCGGCGAACGTGGCGAGAAAGGAAGGGAAGAAAGCGAAAGGAGCGGGCGCTAGGGCGCTGGCAAGTGTAGCGGTCACGCTGCGCGTAACCACCACACCCGCCGCGCTTAATGCGCCGCTACAGGGCGCGTCGCGCCATTCGCCATTCAGGCTGCGCAACTGTTGGGAAGGGCGATCGGTGCGGGCCTCTTCGCTATTACGCCAGCTGGCGAAAGGGGGATGTGCTGCAAGGCGATTAAGTTGGGTAACGCCAGGGTTTTCCCAGTCACGACGTTGTAAAACGACGGCCAGTGAATTGTAATACGACTCACTATAGGGCGAATTGGAGCTCCACCGCGGTGGCGGCCGCTCTAGAATTCCCATTGCATACGTTGTATCCATATCATAATATGTACATTTATATTGGCTCATGTCCAACATTACCGCCATGTTGACATTGATTATTGACTAGTTATTAATAGTAATCAATTACGGGGTCATTAGTTCATAGCCCATATATGGAGTTCCGCGTTACATAACTTACGGTAAATGGCCCGCCTGGCTGACCGCCCAACGACCCCCGCCCATTGACGTCAATAATGACGTATGTTCCCATAGTAACGCCAATAGGGACTTTCCATTGACGTCAATGGGTGGAGTATTTACGGTAAACTGCCCACTTGGCAGTACATCAAGTGTATCATATGCCAAGTACGCCCCCTATTGACGTCAATGACGGTAAATGGCCCGCCTGGCATTATGCCCAGTACATGACCTTATGGGACTTTCCTACTTGGCAGTACATCTACGTATTAGTCATCGCTATTACCATGGTGATGCGGTTTTGGCAGTACATCAATGGGCGTGGATAGCGGTTTGACTCACGGGGATTTCCAAGTCTCCACCCCATTGACGTCAATGGGAGTTTGTTTTGGCACCAAAATCAACGGGACTTTCCAAAATGTCGTAACAACTCCGCCCCATTGACGCAAATGGGCGGTAGGCGTGTACGGTGGGAGGTCTATATAAGCAGAGCTCGTTTAGTGAACCGCAGTCGCTCTGCGGAGAGGCTGGCAGATTGAGCCCTGGGAGGTTCTCTCCAGCACTAGCAGGTAGAGCCTGGGTGTTCCCTGCTAGACTCTCACCAGCACTTGGCCAGTGCTGGGCAGAGTGGCTCCACGCTTGCTTGCTTAAAGACCTCTTCAATAAAGCTGCCATTTTAGAAGTAAGCCAGTGTGTGTTCCCATCTCTCCTAGTCGCCGCCTGGTCAACTCGGTACTCGGTAATAAGAAGACCCTGGTCTGTTAGGACCCTTTCTGCTTTGAGAAACCGAAGCAGGAAAATCCCTAGCAGATTGGCGCCCGAACAGGACTTGAAGGAGAGTGAGAGACTCCTGAGTACGGCTGAGTGAAGGCAGTAAGGGCGGCAGGAACCAACCACGACGGAGTGCTCCTATAAAGGCGCGGGTCGGTACCAGACGGCGTGAGGAGCGGGAGAGGAGGAGGCCTCCGGTTGCAGGTAAGTGCAACACAAAAAAGAAATAGCTGTCTTGTTATCCAGGAAGGGATAATAAGATAGAGTGGGAGATGGGCGCGAGAAACTCCGTCTTGTCAGGGAAGAAAGCAGATGAATTGAAAAAATTATTAATCGCATGAATTTTAAAAGAAGGGGAGGAATAGGGGATATGACTCCAGCAGAAAGATTAATTAACATGATCACTACAGAACAAGAAATACAATTTCAACAATCAAAAAACTCAAAATTTAAAAATTTTCGGGTCTATTACAGAGCTCACGCGTGATTGGAGTTGGGAGATTATAAATTAGTAGAGATCACTCCGATTGGCTTGGCCCCCACAGATGTGAAGAGGTACACTACTGGTGGCACCTCAAGAAATAAAAGAGGGGTCTTTGTGCTAGGGTTCTTGGGTTTTCTCGCAACGGCAGGTTCTGCAATGGGCGCGGCGTCGTTGACGCTGACCGCTCAGTCCCGGACTTTATTGGCTGGGATAGTGCAGCAACAGCAACAGCTGTTGGACGTGGTCAAGAGACAACAAGAATTGTTGCGACTGACCGTCTGGGGAACAAAGAACCTCCAGACTAGGGTCACTGCCATCGAGAAGTACTTAAAGGACCAGGCGCAGCTAAATGCTTGGGGATGTGCGTTTAGACAAGTCTGCCACACTACTGTACCATGGCCAAATGCAAGTCTAACACCAGACTGGAACAATGATACTTGGCAAGAGTGGGAGCGAAAGGTTGACTTCTTGGAGGAAAATATAACAGCCCTCCTAGAAGAGGCACAAATTCAACAAGAGAAGAACATGTATGAATTACAAAAGTTGAATAGCTGGGATGTGTTTGGCAATTGGTTTGACCTTGCTTCTTGGATAAAGTATATACAATATGGAATTTATGTAGTTGTAGGAGTAATACTGTTAAGAATAGTGATCTATATAGTACAAATGCTAGCTAAGTTAAGGCAGGGGTATAGGCCAGTGTTCTCTTCCCCACCCTCTTATTTCCAGTAGACTCATACCCAACAGGACCCGGCACTGCCAACCAGAGAAGGCAAAGAAGGAGACGGTGGAGAAGGCGGTGGATCCTATTATCGATCCATTGCATACGTTGTATCCATATCATAATATGTACATTTATATTGGCTCATGTCCAACATTACCGCCATGTTGACATTGATTATTGACTAGTTATTAATAGTAATCAATTACGGGGTCATTAGTTCATAGCCCATATATGGAGTTCCGCGTTACATAACTTACGGTAAATGGCCCGCCTGGCTGACCGCCCAACGACCCCCGCCCATTGACGTCAATAATGACGTATGTTCCCATAGTAACGCCAATAGGGACTTTCCATTGACGTCAATGGGTGGAGTATTTACGGTAAACTGCCCACTTGGCAGTACATCAAGTGTATCATATGCCAAGTACGCCCCCTATTGACGTCAATGACGGTAAATGGCCCGCCTGGCATTATGCCCAGTACATGACCTTATGGGACTTTCCTACTTGGCAGTACATCTACGTATTAGTCATCGCTATTACCATGGTGATGCGGTTTTGGCAGTACgTCAATGGGCGTGGATAGCGGTTTGACTCACGGGGATTTCCAAGTCTCCACCCCATTGACGTCAATGGGAGTTTGTTTTGGCACCAAAATCAACGGGACTTTCCAAAATGTCGTAACAACTCCGCCCCATTGACGCAAATGGGCGGTAGGCGTGTACGGTGGGAGGTCTATATAAGCAGAGCTCGTTTAGTGAACCGTCAGATCCAGCTGGCTaccggtACCATGGGTCCAAAAAAGAAGAGAAAGGTAGATCCAAAAAAGAAGAGAAAGGTAGATCCAAAAAAGAAGAGAAAGGTAATGAGGGGCAGCCACCACCACCACCACCACGGCAGCCTGGCCACCACCCTGGAGAGGATCGAGAAGAACTTCGTGATCACCGACCCCAGGCTGCCCGACAACCCCATCATCTTCGCCAGCGACAGCTTCCTGCAGCTGACCGAGTACAGCAGGGAGGAGATCCTGGGCAGGAACTGCAGGTTCCTGCAGGGCCCCGAGACCGACAGGGCCACCGTGAGGAAGATCAGGGACGCCATCGACAACCAGACCGAGGTGACCGTGCAGCTGATCAACTACACCAAGAGCGGCAAGAAGTTCTGGAACGTGTTCCACCTGCAGCCCATGAGGGACTACAAGGGCGACGTGCAGTACTTCATCGGCGTGCAGCTGGACGGCACCGAGAGGCTGCACGGCGCCGCCGAGAGGGAGGCCGTGTGCCTGATCAAGAAGACCGCCTTCCAGATCGACAGCGACGAGGTGAGGAAGAACCTGATGGACATGTTCAGGGACAGGCAGGCCTTCAGCGAGCACACCTGGAAGATGCTGCTGAGCGTGTGCAGGAGCTGGGCCGCCTGGTGCAAGCTGAACAACAGGAAGTGGTTCCCCGCCGAGCCCGAGGACGTGAGGGACTACCTGCTGTACCTGCAGGCCAGGGGCCTGGCCGTGAAGACCATCCAGCAGCACCTGGGCCAGCTGAACATGCTGCACAGGAGGAGCGGCCTGCCCAGGCCCAGCGACAGCAACGCCGTGAGCCTGGTGATGAGGAGGATCAGGAAGGAGAACGTGGACGCCGGCGAGAGGGCCAAGCAGGCCCTGGCCTTCGAGAGGACCGACTTCGACCAGGTGAGGAGCCTGATGGAGAACAGCGACAGGTGCCAGGACATCAGGAACCTGGCCTTCCTGGGCATCGCCTACAACACCCTGCTGAGGATCGCCGAGATCGCCAGGATCAGGGTGAAGGACATCAGCAGGACCGACGGCGGCAGGATGCTGATCCACATCGGCAGGACCAAGACCCTGGTGAGCACCGCCGGCGTGGAGAAGGCCCTGAGCCTGGGCGTGACCAAGCTGGTGGAGAGGTGGATCAGCGTGAGCGGCGTGGCCGACGACCCCAACAACTACCTGTTCTGCAGGGTGAGGAAGAACGGCGTGGCCGCCCCCAGCGCCACCAGCCAGCTGAGCACCAGGGCCCTGGAGGGCATCTTCGAGGCCACCCACAGGCTGATCTACGGCGCCAAGGACGACAGCGGCCAGAGGTACCTGGCCTGGAGCGGCCACAGCGCCAGGGTGGGCGCCGCCAGGGACATGGCCAGGGCCGGCGTGAGCATCCCCGAGATCATGCAGGCCGGCGGCTGGACCAACGTGAACATCGTGATGAACTACATCAGGAACCTGGACAGCGAGACCGGCGCCATGGTGAGGCTGCTGGCCGCCGGCGACTAAGCTTGGATCCAATCAACCTCTGGATTACAAAATTTGTGAAAGATTGACTGGTATTCTTAACTATGTTGCTCCTTTTACGCTATGTGGATACGCTGCTTTAATGCCTTTGTATCATGCTATTGCTTCCCGTATGGCTTTCATTTTCTCCTCCTTGTATAAATCCTGGTTGCTGTCTCTTTATGAGGAGTTGTGGCCCGTTGTCAGGCAACGTGGCGTGGTGTGCACTGTGTTTGCTGACGCAACCCCCACTGGTTGGGGCATTGCCACCACCTGTCAGCTCCTTTCCGGGACTTTCGCTTTCCCCCTCCCTATTGCCACGGCGGAACTCATCGCCGCCTGCCTTGCCCGCTGCTGGACAGGGGCTCGGCTGTTGGGCACTGACAATTCCGTGGTGTTGTCGGGGAAGCTGACGTCCTTTCCATGGCTGCTCGCCTGTGTTGCCACCTGGATTCTGCGCGGGACGTCCTTCTGCTACGTCCCTTCGGCCCTCAATCCAGCGGACCTTCCTTCCCGCGGCCTGCTGCCGGCTCTGCGGCCTCTTCCGCGTCTTCGCCTTCGCCCTCAGACGAGTCGGATCTCCCTTTGGGCCGCCTCCCCGCCTGtttCGCCTCGCGACTCGAGCAAaCTcACaCTcAAGTTtATtTGCAAaCTcACaCTcAAGTTtATtTGCAAaCTcACaCTcAAGTCATGCATGgTCGAGTTTTATAAAAGAAAAGGGGGGACTGGAAGGGATTTATTACAGTGCAAGAAGACATAGAATCTTAGACATGTACTTAGAAAAGGAAGAAGGCATCATACCAGATTGGCAGGATTACACCTCAGGACCAGGAATTAGATACCCAAAGACATTTGGCTGGCTATGGAAATTAGTCCCTGTAAATGTATCAGATGAGGCACAGGAGGATGAGAGGCATTATTTAATGCAGCCAGCTCAAACTTCCAAGTGGGATGACCCTTGGGGAGAGGTTCTAGCGTGGAAGTTTGATCCAACTCTAGCCTACACTTATGAGGCATATGCTAGATACCCAGAAGAGTTGGAAGCAAGTCAGGCCTGTCAGAACTGCATTTCGCTCTGTATTCAGTCGCTCTGCGGAGAGGCTGGCAGATTGAGCCCTGGGAGGTTCTCTCCAGCACTAGCAGGTAGAGCCTGGGTGTTCCCTGCTAGACTCTCACCAGCACTTGGCCAGTGCTGGGCAGAGTGGCTCCACGCTTGCTTGCTTAAAGACCTCTTCAATAAAGCTGCCATTTTAGAAGTAAGCCAGTGTGTGTTCCCATCTCTCCTAGTCGCCGCCTGGTCAACTCGGTACTCGGTAATAAGAAGACCCTGGTCTGTTAGGACCCTTTCTGCTTTGAGAAACCGAAGCAGGAAAATCCCTAGCATGGTACCCAGCTTTTGTTCCCTTTAGTGAGGGTTAATTCCGAGCTTGGCGTAATCATGGTCATAGCTGTTTCCTGTGTGAAATTGTTATCCGCTCACAATTCCACACAACATACGAGCCGGAAGCATAAAGTGTAAAGCCTGGGGTGCCTAATGAGTGAGCTAACTCACATTAATTGCGTTGCGCTCACTGCCCGCTTTCCAGTCGGGAAACCTGTCGTGCCAGCTGCATTAATGAATCGGCCAACGCGCGGGGAGAGGCGGTTTGCGTATTGGGCGCTCTTCCGCTTCCTCGCTCACTGACTCGCTGCGCTCGGTCGTTCGGCTGCGGCGAGCGGTATCAGCTCACTCAAAGGCGGTAATACGGTTATCCACAGAATCAGGGGATAACGCAGGAAAGAACATGTGAGCAAAAGGCCAGCAAAAGGCCAGGAACCGTAAAAAGGCCGCGTTGCTGGCGTTTTTCCATAGGCTCCGCCCCCCTGACGAGCATCACAAAAATCGACGCTCAAGTCAGAGGTGGCGAAACCCGACAGGACTATAAAGATACCAGGCGTTTCCCCCTGGAAGCTCCCTCGTGCGCTCTCCTGTTCCGACCCTGCCGCTTACCGGATACCTGTCCGCCTTTCTCCCTTCGGGAAGCGTGGCGCTTTCTCATAGCTCACGCTGTAGGTATCTCAGTTCGGTGTAGGTCGTTCGCTCCAAGCTGGGCTGTGTGCACGAACCCCCCGTTCAGCCCGACCGCTGCGCCTTATCCGGTAACTATCGTCTTGAGTCCAACCCGGTAAGACACGACTTATCGCCACTGGCAGCAGCCACTGGTAACAGGATTAGCAGAGCGAGGTATGTAGGCGGTGCTACAGAGTTCTTGAAGTGGTGGCCTAACTACGGCTACACTAGAAGGACAGTATTTGGTATCTGCGCTCTGCTGAAGCCAGTTACCTTCGGAAAAAGAGTTGGTAGCTCTTGATCCGGCAAACAAACCACCGCTGGTAGCGGTGGTTTTTTTGTTTGCAAGCAGCAGATTACGCGCAGAAAAAAAGGATCTCAAGAAGATCCTTTGATCTTTTCTACGGGGTCTGACGCTCAGTGGAACGAAAACTCACGTTAAGGGATTTTGGTCATGAGATTATCAAAAAGGATCTTCACCTAGATCCTTTTAAATTAAAAATGAAGTTTTAAATCAATCTAAAGTATATATGAGTAAACTTGGTCTGACAGTTACCAATGCTTAATCAGTGAGGCACCTATCTCAGCGATCTGTCTATTTCGTTCATCCATAGTTGCCTGACTCCCCGTCGTGTAGATAACTACGATACGGGAGGGCTTACCATCTGGCCCCAGTGCTGCAATGATACCGCGAGACCCACGCTCACCGGCTCCAGATTTATCAGCAATAAACCAGCCAGCCGGAAGGGCCGAGCGCAGAAGTGGTCCTGCAACTTTATCCGCCTCCATCCAGTCTATTAATTGTTGCCGGGAAGCTAGAGTAAGTAGTTCGCCAGTTAATAGTTTGCGCAACGTTGTTGCCATTGCTACAGGCATCGTGGTGTCACGCTCGTCGTTTGGTATGGCTTCATTCAGCTCCGGTTCCCAACGATCAAGGCGAGTTACATGATCCCCCATGTTGTGCAAAAAAGCGGTTAGCTCCTTCGGTCCTCCGATCGTTGTCAGAAGTAAGTTGGCCGCAGTGTTATCACTCATGGTTATGGCAGCACTGCATAATTCTCTTACTGTCATGCCATCCGTAAGATGCTTTTCTGTGACTGGTGAGTACTCAACCAAGTCATTCTGAGAATAGTGTATGCGGCGACCGAGTTGCTCTTGCCCGGCGTCAATACGGGATAATACCGCGCCACATAGCAGAACTTTAAAAGTGCTCATCATTGGAAAACGTTCTTCGGGGCGAAAACTCTCAAGGATCTTACCGCTGTTGAGATCCAGTTCGATGTAACCCACTCGTGCACCCAACTGATCTTCAGCATCTTTTACTTTCACCAGCGTTTCTGGGTGAGCAAAAACAGGAAGGCAAAATGCCGCAAAAAAGGGAATAAGGGCGACACGGAAATGTTGAATACTCATACTCTTCCTTTTTCAATATTATTGAAGCATTTATCAGGGTTATTGTCTCATGAGCGGATACATATTTGAATGTATTTAGAAAAATAAACAAATAGGGGTTCCGCGCACATTTCCCCGAAAAGTGCCACCTG

>pGY625

GGAAATTGTAAACGTTAATATTTTGTTAAAATTCGCGTTAAATTTTTGTTAAATCAGCTCATTTTTTAACCAATAGGCCGAAATCGGCAAAATCCCTTATAAATCAAAAGAATAGACCGAGATAGGGTTGAGTGTTGTTCCAGTTTGGAACAAGAGTCCACTATTAAAGAACGTGGACTCCAACGTCAAAGGGCGAAAAACCGTCTATCAGGGCGATGGCCCACTACGTGAACCATCACCCTAATCAAGTTTTTTGGGGTCGAGGTGCCGTAAAGCACTAAATCGGAACCCTAAAGGGAGCCCCCGATTTAGAGCTTGACGGGGAAAGCCGGCGAACGTGGCGAGAAAGGAAGGGAAGAAAGCGAAAGGAGCGGGCGCTAGGGCGCTGGCAAGTGTAGCGGTCACGCTGCGCGTAACCACCACACCCGCCGCGCTTAATGCGCCGCTACAGGGCGCGTCGCGCCATTCGCCATTCAGGCTGCGCAACTGTTGGGAAGGGCGATCGGTGCGGGCCTCTTCGCTATTACGCCAGCTGGCGAAAGGGGGATGTGCTGCAAGGCGATTAAGTTGGGTAACGCCAGGGTTTTCCCAGTCACGACGTTGTAAAACGACGGCCAGTGAATTGTAATACGACTCACTATAGGGCGAATTGGAGCTCCACCGCGGTGGCGGCCGCTCTAGAATTCCCATTGCATACGTTGTATCCATATCATAATATGTACATTTATATTGGCTCATGTCCAACATTACCGCCATGTTGACATTGATTATTGACTAGTTATTAATAGTAATCAATTACGGGGTCATTAGTTCATAGCCCATATATGGAGTTCCGCGTTACATAACTTACGGTAAATGGCCCGCCTGGCTGACCGCCCAACGACCCCCGCCCATTGACGTCAATAATGACGTATGTTCCCATAGTAACGCCAATAGGGACTTTCCATTGACGTCAATGGGTGGAGTATTTACGGTAAACTGCCCACTTGGCAGTACATCAAGTGTATCATATGCCAAGTACGCCCCCTATTGACGTCAATGACGGTAAATGGCCCGCCTGGCATTATGCCCAGTACATGACCTTATGGGACTTTCCTACTTGGCAGTACATCTACGTATTAGTCATCGCTATTACCATGGTGATGCGGTTTTGGCAGTACATCAATGGGCGTGGATAGCGGTTTGACTCACGGGGATTTCCAAGTCTCCACCCCATTGACGTCAATGGGAGTTTGTTTTGGCACCAAAATCAACGGGACTTTCCAAAATGTCGTAACAACTCCGCCCCATTGACGCAAATGGGCGGTAGGCGTGTACGGTGGGAGGTCTATATAAGCAGAGCTCGTTTAGTGAACCGCAGTCGCTCTGCGGAGAGGCTGGCAGATTGAGCCCTGGGAGGTTCTCTCCAGCACTAGCAGGTAGAGCCTGGGTGTTCCCTGCTAGACTCTCACCAGCACTTGGCCAGTGCTGGGCAGAGTGGCTCCACGCTTGCTTGCTTAAAGACCTCTTCAATAAAGCTGCCATTTTAGAAGTAAGCCAGTGTGTGTTCCCATCTCTCCTAGTCGCCGCCTGGTCAACTCGGTACTCGGTAATAAGAAGACCCTGGTCTGTTAGGACCCTTTCTGCTTTGAGAAACCGAAGCAGGAAAATCCCTAGCAGATTGGCGCCCGAACAGGACTTGAAGGAGAGTGAGAGACTCCTGAGTACGGCTGAGTGAAGGCAGTAAGGGCGGCAGGAACCAACCACGACGGAGTGCTCCTATAAAGGCGCGGGTCGGTACCAGACGGCGTGAGGAGCGGGAGAGGAGGAGGCCTCCGGTTGCAGGTAAGTGCAACACAAAAAAGAAATAGCTGTCTTGTTATCCAGGAAGGGATAATAAGATAGAGTGGGAGATGGGCGCGAGAAACTCCGTCTTGTCAGGGAAGAAAGCAGATGAATTGAAAAAATTATTAATCGCATGAATTTTAAAAGAAGGGGAGGAATAGGGGATATGACTCCAGCAGAAAGATTAATTAACATGATCACTACAGAACAAGAAATACAATTTCAACAATCAAAAAACTCAAAATTTAAAAATTTTCGGGTCTATTACAGAGCTCACGCGTGATTGGAGTTGGGAGATTATAAATTAGTAGAGATCACTCCGATTGGCTTGGCCCCCACAGATGTGAAGAGGTACACTACTGGTGGCACCTCAAGAAATAAAAGAGGGGTCTTTGTGCTAGGGTTCTTGGGTTTTCTCGCAACGGCAGGTTCTGCAATGGGCGCGGCGTCGTTGACGCTGACCGCTCAGTCCCGGACTTTATTGGCTGGGATAGTGCAGCAACAGCAACAGCTGTTGGACGTGGTCAAGAGACAACAAGAATTGTTGCGACTGACCGTCTGGGGAACAAAGAACCTCCAGACTAGGGTCACTGCCATCGAGAAGTACTTAAAGGACCAGGCGCAGCTAAATGCTTGGGGATGTGCGTTTAGACAAGTCTGCCACACTACTGTACCATGGCCAAATGCAAGTCTAACACCAGACTGGAACAATGATACTTGGCAAGAGTGGGAGCGAAAGGTTGACTTCTTGGAGGAAAATATAACAGCCCTCCTAGAAGAGGCACAAATTCAACAAGAGAAGAACATGTATGAATTACAAAAGTTGAATAGCTGGGATGTGTTTGGCAATTGGTTTGACCTTGCTTCTTGGATAAAGTATATACAATATGGAATTTATGTAGTTGTAGGAGTAATACTGTTAAGAATAGTGATCTATATAGTACAAATGCTAGCTAAGTTAAGGCAGGGGTATAGGCCAGTGTTCTCTTCCCCACCCTCTTATTTCCAGTAGACTCATACCCAACAGGACCCGGCACTGCCAACCAGAGAAGGCAAAGAAGGAGACGGTGGAGAAGGCGGTGGATCCTATTATCGATCCATTGCATACGTTGTATCCATATCATAATATGTACATTTATATTGGCTCATGTCCAACATTACCGCCATGTTGACATTGATTATTGACTAGTTATTAATAGTAATCAATTACGGGGTCATTAGTTCATAGCCCATATATGGAGTTCCGCGTTACATAACTTACGGTAAATGGCCCGCCTGGCTGACCGCCCAACGACCCCCGCCCATTGACGTCAATAATGACGTATGTTCCCATAGTAACGCCAATAGGGACTTTCCATTGACGTCAATGGGTGGAGTATTTACGGTAAACTGCCCACTTGGCAGTACATCAAGTGTATCATATGCCAAGTACGCCCCCTATTGACGTCAATGACGGTAAATGGCCCGCCTGGCATTATGCCCAGTACATGACCTTATGGGACTTTCCTACTTGGCAGTACATCTACGTATTAGTCATCGCTATTACCATGGTGATGCGGTTTTGGCAGTACgTCAATGGGCGTGGATAGCGGTTTGACTCACGGGGATTTCCAAGTCTCCACCCCATTGACGTCAATGGGAGTTTGTTTTGGCACCAAAATCAACGGGACTTTCCAAAATGTCGTAACAACTCCGCCCCATTGACGCAAATGGGCGGTAGGCGTGTACGGTGGGAGGTCTATATAAGCAGAGCTCGTTTAGTGAACCGTCAGATCCAGCTGGCTaccggtACCATGGCCACCTCTGATGAAGTCAGGAAGAACCTGATGGACATGTTCAGGGACAGGCAGGCCTTCTCTGAACACACCTGGAAGATGCTCCTGTCTGTGTGCAGATCCTGGGCTGCCTGGTGCAAGCTGAACGGTACCCATACTCTTTATGCCCCCGGTGGATATGACATTATGGGATAcCTGGACCAGATCGGCAACCGGCCAAACCCGCAGGTGGAACTGGGCCCCGTGGATACATCCTGCGCCTTGATTCTTTGTGACCTGAAACAGAAAGACACCCCGATAGTTTACGCGAGTGAAGCCTTCCTCTACATGACAGGTTACAGCAACGCAGAGGTGCTGGGCCGGAATTGCCGGTTTCTGCAAAGCCCTGACGGCATGGTGAAGCCCAAGAGCACCCGGAAGTACGTGGATAGTAACACAATCAATACTATGCGCAAGGCAATCGACAGGAATGCCGAGGTGCAGGTTGAAGTAGTCAATTTTAAAAAGAATGGACAGCGATTTGTTAATTTCCTGACTATGATACCTGTTAGGGACGAAACAGGCGAGTATCGATACTCTATGGGATTCCAGTGCGAAACAGAAGGCGGAAGCGGTGGCGTGCCCAAGAAGAAGAGGAAAGTCGGATCCGGCAGCGGCGCCACCAACTTCAGCCTGCTGAAGCAGGCCGGCGACGTGGAGGAGAACCCCGGCCCCCTCGAGGTGCCCAAGAAGAAGAGGAAAGTCGGCGGACATACTCTTTATGCCCCCGGTGGATATGACATTATGGGATATCTGAGGCAGATCAGGAACCGGCCAAACCCGCAGGTGGAACTGGGCCCCGTGGATACATCCTGCGCCTTGATTCTTTGTGACCTGAAACAGAAAGACACCCCGATAGTTTACGCGAGTGAAGCCTTCCTCTACATGACAGGTTACAGCAACGCAGAGGTGCTGGGCCGGAATTGCCGGTTTCTGCAAAGCCCTGACGGCATGGTGAAGCCCAAGAGCACCCGGAAGTACGTGGATAGTAACACAATCAATACTATGCGCAAGGCAATCGACAGGAATGCCGAGGTGCAGGTTGAAGTAGTCAATTTTAAAAAGAATGGACAGCGATTTGTTAATTTCCTGACTATGATACCTGTTAGGGACGAAACAGGCGAGTATCGATACTCTATGGGATTCCAGTGCGAAACAGAAGGTACCAACAGGAAATGGTTCCCTGCTGAACCTGAGGATGTGAGGGACTACCTCCTGTACCTGCAAGCCAGAGGCCTGGCTGTGAAGACCATCCAACAGCACCTGGGCCAGCTCAACATGCTGCACAGGAGATCTGGCCTGCCTCGCCCTTCTGACTCCAATGCTGTGTCCCTGGTGATGAGGAGAATCAGAAAGGAGAATGTGGATGCTGGGGAGAGAGCCAAGCAGGCCCTGGCCTTTGAACGCACTGACTTTGACCAAGTCAGATCCCTGATGGAGAACTCTGACAGATGCCAGGACATCAGGAACCTGGCCTTCCTGGGCATTGCCTACAACACCCTGCTGCGCATTGCCGAAATTGCCAGAATCAGAGTGAAGGACATCTCCCGCACCGATGGTGGGAGAATGCTGATCCACATTGGCAGGACCAAGACCCTGGTGTCCACAGCTGGTGTGGAGAAGGCCCTGTCCCTGGGGGTTACCAAGCTGGTGGAGAGATGGATCTCTGTGTCTGGTGTGGCTGATGACCCCAACAACTACCTGTTCTGCCGGGTCAGAAAGAATGGTGTGGCTGCCCCTTCTGCCACCTCCCAACTGTCCACCCGGGCCCTGGAAGGGATCTTTGAGGCCACCCACCGCCTGATCTATGGTGCCAAGGATGACTCTGGGCAGAGATACCTGGCCTGGTCTGGCCACTCTGCCAGAGTGGGTGCTGCCAGGGACATGGCCAGGGCTGGTGTGTCCATCCCTGAAATCATGCAGGCTGGTGGCTGGACCAATGTGAACATTGTGATGAACTACATCAGAAACCTGGACTCTGAGACTGGGGCCATGGTGAGGCTGCTCGAAGATGGGGACTAAGCTTGGATCCAATCAACCTCTGGATTACAAAATTTGTGAAAGATTGACTGGTATTCTTAACTATGTTGCTCCTTTTACGCTATGTGGATACGCTGCTTTAATGCCTTTGTATCATGCTATTGCTTCCCGTATGGCTTTCATTTTCTCCTCCTTGTATAAATCCTGGTTGCTGTCTCTTTATGAGGAGTTGTGGCCCGTTGTCAGGCAACGTGGCGTGGTGTGCACTGTGTTTGCTGACGCAACCCCCACTGGTTGGGGCATTGCCACCACCTGTCAGCTCCTTTCCGGGACTTTCGCTTTCCCCCTCCCTATTGCCACGGCGGAACTCATCGCCGCCTGCCTTGCCCGCTGCTGGACAGGGGCTCGGCTGTTGGGCACTGACAATTCCGTGGTGTTGTCGGGGAAGCTGACGTCCTTTCCATGGCTGCTCGCCTGTGTTGCCACCTGGATTCTGCGCGGGACGTCCTTCTGCTACGTCCCTTCGGCCCTCAATCCAGCGGACCTTCCTTCCCGCGGCCTGCTGCCGGCTCTGCGGCCTCTTCCGCGTCTTCGCCTTCGCCCTCAGACGAGTCGGATCTCCCTTTGGGCCGCCTCCCCGCCTGtttCGCCTCGCGACTCGAGCAAaCTcACaCTcAAGTTtATtTGCAAaCTcACaCTcAAGTTtATtTGCAAaCTcACaCTcAAGTCATGCATGgTCGAGTTTTATAAAAGAAAAGGGGGGACTGGAAGGGATTTATTACAGTGCAAGAAGACATAGAATCTTAGACATGTACTTAGAAAAGGAAGAAGGCATCATACCAGATTGGCAGGATTACACCTCAGGACCAGGAATTAGATACCCAAAGACATTTGGCTGGCTATGGAAATTAGTCCCTGTAAATGTATCAGATGAGGCACAGGAGGATGAGAGGCATTATTTAATGCAGCCAGCTCAAACTTCCAAGTGGGATGACCCTTGGGGAGAGGTTCTAGCGTGGAAGTTTGATCCAACTCTAGCCTACACTTATGAGGCATATGCTAGATACCCAGAAGAGTTGGAAGCAAGTCAGGCCTGTCAGAACTGCATTTCGCTCTGTATTCAGTCGCTCTGCGGAGAGGCTGGCAGATTGAGCCCTGGGAGGTTCTCTCCAGCACTAGCAGGTAGAGCCTGGGTGTTCCCTGCTAGACTCTCACCAGCACTTGGCCAGTGCTGGGCAGAGTGGCTCCACGCTTGCTTGCTTAAAGACCTCTTCAATAAAGCTGCCATTTTAGAAGTAAGCCAGTGTGTGTTCCCATCTCTCCTAGTCGCCGCCTGGTCAACTCGGTACTCGGTAATAAGAAGACCCTGGTCTGTTAGGACCCTTTCTGCTTTGAGAAACCGAAGCAGGAAAATCCCTAGCATGGTACCCAGCTTTTGTTCCCTTTAGTGAGGGTTAATTCCGAGCTTGGCGTAATCATGGTCATAGCTGTTTCCTGTGTGAAATTGTTATCCGCTCACAATTCCACACAACATACGAGCCGGAAGCATAAAGTGTAAAGCCTGGGGTGCCTAATGAGTGAGCTAACTCACATTAATTGCGTTGCGCTCACTGCCCGCTTTCCAGTCGGGAAACCTGTCGTGCCAGCTGCATTAATGAATCGGCCAACGCGCGGGGAGAGGCGGTTTGCGTATTGGGCGCTCTTCCGCTTCCTCGCTCACTGACTCGCTGCGCTCGGTCGTTCGGCTGCGGCGAGCGGTATCAGCTCACTCAAAGGCGGTAATACGGTTATCCACAGAATCAGGGGATAACGCAGGAAAGAACATGTGAGCAAAAGGCCAGCAAAAGGCCAGGAACCGTAAAAAGGCCGCGTTGCTGGCGTTTTTCCATAGGCTCCGCCCCCCTGACGAGCATCACAAAAATCGACGCTCAAGTCAGAGGTGGCGAAACCCGACAGGACTATAAAGATACCAGGCGTTTCCCCCTGGAAGCTCCCTCGTGCGCTCTCCTGTTCCGACCCTGCCGCTTACCGGATACCTGTCCGCCTTTCTCCCTTCGGGAAGCGTGGCGCTTTCTCATAGCTCACGCTGTAGGTATCTCAGTTCGGTGTAGGTCGTTCGCTCCAAGCTGGGCTGTGTGCACGAACCCCCCGTTCAGCCCGACCGCTGCGCCTTATCCGGTAACTATCGTCTTGAGTCCAACCCGGTAAGACACGACTTATCGCCACTGGCAGCAGCCACTGGTAACAGGATTAGCAGAGCGAGGTATGTAGGCGGTGCTACAGAGTTCTTGAAGTGGTGGCCTAACTACGGCTACACTAGAAGGACAGTATTTGGTATCTGCGCTCTGCTGAAGCCAGTTACCTTCGGAAAAAGAGTTGGTAGCTCTTGATCCGGCAAACAAACCACCGCTGGTAGCGGTGGTTTTTTTGTTTGCAAGCAGCAGATTACGCGCAGAAAAAAAGGATCTCAAGAAGATCCTTTGATCTTTTCTACGGGGTCTGACGCTCAGTGGAACGAAAACTCACGTTAAGGGATTTTGGTCATGAGATTATCAAAAAGGATCTTCACCTAGATCCTTTTAAATTAAAAATGAAGTTTTAAATCAATCTAAAGTATATATGAGTAAACTTGGTCTGACAGTTACCAATGCTTAATCAGTGAGGCACCTATCTCAGCGATCTGTCTATTTCGTTCATCCATAGTTGCCTGACTCCCCGTCGTGTAGATAACTACGATACGGGAGGGCTTACCATCTGGCCCCAGTGCTGCAATGATACCGCGAGACCCACGCTCACCGGCTCCAGATTTATCAGCAATAAACCAGCCAGCCGGAAGGGCCGAGCGCAGAAGTGGTCCTGCAACTTTATCCGCCTCCATCCAGTCTATTAATTGTTGCCGGGAAGCTAGAGTAAGTAGTTCGCCAGTTAATAGTTTGCGCAACGTTGTTGCCATTGCTACAGGCATCGTGGTGTCACGCTCGTCGTTTGGTATGGCTTCATTCAGCTCCGGTTCCCAACGATCAAGGCGAGTTACATGATCCCCCATGTTGTGCAAAAAAGCGGTTAGCTCCTTCGGTCCTCCGATCGTTGTCAGAAGTAAGTTGGCCGCAGTGTTATCACTCATGGTTATGGCAGCACTGCATAATTCTCTTACTGTCATGCCATCCGTAAGATGCTTTTCTGTGACTGGTGAGTACTCAACCAAGTCATTCTGAGAATAGTGTATGCGGCGACCGAGTTGCTCTTGCCCGGCGTCAATACGGGATAATACCGCGCCACATAGCAGAACTTTAAAAGTGCTCATCATTGGAAAACGTTCTTCGGGGCGAAAACTCTCAAGGATCTTACCGCTGTTGAGATCCAGTTCGATGTAACCCACTCGTGCACCCAACTGATCTTCAGCATCTTTTACTTTCACCAGCGTTTCTGGGTGAGCAAAAACAGGAAGGCAAAATGCCGCAAAAAAGGGAATAAGGGCGACACGGAAATGTTGAATACTCATACTCTTCCTTTTTCAATATTATTGAAGCATTTATCAGGGTTATTGTCTCATGAGCGGATACATATTTGAATGTATTTAGAAAAATAAACAAATAGGGGTTCCGCGCACATTTCCCCGAAAAGTGCCACCTG

>pGY626

GGAAATTGTAAACGTTAATATTTTGTTAAAATTCGCGTTAAATTTTTGTTAAATCAGCTCATTTTTTAACCAATAGGCCGAAATCGGCAAAATCCCTTATAAATCAAAAGAATAGACCGAGATAGGGTTGAGTGTTGTTCCAGTTTGGAACAAGAGTCCACTATTAAAGAACGTGGACTCCAACGTCAAAGGGCGAAAAACCGTCTATCAGGGCGATGGCCCACTACGTGAACCATCACCCTAATCAAGTTTTTTGGGGTCGAGGTGCCGTAAAGCACTAAATCGGAACCCTAAAGGGAGCCCCCGATTTAGAGCTTGACGGGGAAAGCCGGCGAACGTGGCGAGAAAGGAAGGGAAGAAAGCGAAAGGAGCGGGCGCTAGGGCGCTGGCAAGTGTAGCGGTCACGCTGCGCGTAACCACCACACCCGCCGCGCTTAATGCGCCGCTACAGGGCGCGTCGCGCCATTCGCCATTCAGGCTGCGCAACTGTTGGGAAGGGCGATCGGTGCGGGCCTCTTCGCTATTACGCCAGCTGGCGAAAGGGGGATGTGCTGCAAGGCGATTAAGTTGGGTAACGCCAGGGTTTTCCCAGTCACGACGTTGTAAAACGACGGCCAGTGAATTGTAATACGACTCACTATAGGGCGAATTGGAGCTCCACCGCGGTGGCGGCCGCTCTAGAATTCCCATTGCATACGTTGTATCCATATCATAATATGTACATTTATATTGGCTCATGTCCAACATTACCGCCATGTTGACATTGATTATTGACTAGTTATTAATAGTAATCAATTACGGGGTCATTAGTTCATAGCCCATATATGGAGTTCCGCGTTACATAACTTACGGTAAATGGCCCGCCTGGCTGACCGCCCAACGACCCCCGCCCATTGACGTCAATAATGACGTATGTTCCCATAGTAACGCCAATAGGGACTTTCCATTGACGTCAATGGGTGGAGTATTTACGGTAAACTGCCCACTTGGCAGTACATCAAGTGTATCATATGCCAAGTACGCCCCCTATTGACGTCAATGACGGTAAATGGCCCGCCTGGCATTATGCCCAGTACATGACCTTATGGGACTTTCCTACTTGGCAGTACATCTACGTATTAGTCATCGCTATTACCATGGTGATGCGGTTTTGGCAGTACATCAATGGGCGTGGATAGCGGTTTGACTCACGGGGATTTCCAAGTCTCCACCCCATTGACGTCAATGGGAGTTTGTTTTGGCACCAAAATCAACGGGACTTTCCAAAATGTCGTAACAACTCCGCCCCATTGACGCAAATGGGCGGTAGGCGTGTACGGTGGGAGGTCTATATAAGCAGAGCTCGTTTAGTGAACCGCAGTCGCTCTGCGGAGAGGCTGGCAGATTGAGCCCTGGGAGGTTCTCTCCAGCACTAGCAGGTAGAGCCTGGGTGTTCCCTGCTAGACTCTCACCAGCACTTGGCCAGTGCTGGGCAGAGTGGCTCCACGCTTGCTTGCTTAAAGACCTCTTCAATAAAGCTGCCATTTTAGAAGTAAGCCAGTGTGTGTTCCCATCTCTCCTAGTCGCCGCCTGGTCAACTCGGTACTCGGTAATAAGAAGACCCTGGTCTGTTAGGACCCTTTCTGCTTTGAGAAACCGAAGCAGGAAAATCCCTAGCAGATTGGCGCCCGAACAGGACTTGAAGGAGAGTGAGAGACTCCTGAGTACGGCTGAGTGAAGGCAGTAAGGGCGGCAGGAACCAACCACGACGGAGTGCTCCTATAAAGGCGCGGGTCGGTACCAGACGGCGTGAGGAGCGGGAGAGGAGGAGGCCTCCGGTTGCAGGTAAGTGCAACACAAAAAAGAAATAGCTGTCTTGTTATCCAGGAAGGGATAATAAGATAGAGTGGGAGATGGGCGCGAGAAACTCCGTCTTGTCAGGGAAGAAAGCAGATGAATTGAAAAAATTATTAATCGCATGAATTTTAAAAGAAGGGGAGGAATAGGGGATATGACTCCAGCAGAAAGATTAATTAACATGATCACTACAGAACAAGAAATACAATTTCAACAATCAAAAAACTCAAAATTTAAAAATTTTCGGGTCTATTACAGAGCTCACGCGTGATTGGAGTTGGGAGATTATAAATTAGTAGAGATCACTCCGATTGGCTTGGCCCCCACAGATGTGAAGAGGTACACTACTGGTGGCACCTCAAGAAATAAAAGAGGGGTCTTTGTGCTAGGGTTCTTGGGTTTTCTCGCAACGGCAGGTTCTGCAATGGGCGCGGCGTCGTTGACGCTGACCGCTCAGTCCCGGACTTTATTGGCTGGGATAGTGCAGCAACAGCAACAGCTGTTGGACGTGGTCAAGAGACAACAAGAATTGTTGCGACTGACCGTCTGGGGAACAAAGAACCTCCAGACTAGGGTCACTGCCATCGAGAAGTACTTAAAGGACCAGGCGCAGCTAAATGCTTGGGGATGTGCGTTTAGACAAGTCTGCCACACTACTGTACCATGGCCAAATGCAAGTCTAACACCAGACTGGAACAATGATACTTGGCAAGAGTGGGAGCGAAAGGTTGACTTCTTGGAGGAAAATATAACAGCCCTCCTAGAAGAGGCACAAATTCAACAAGAGAAGAACATGTATGAATTACAAAAGTTGAATAGCTGGGATGTGTTTGGCAATTGGTTTGACCTTGCTTCTTGGATAAAGTATATACAATATGGAATTTATGTAGTTGTAGGAGTAATACTGTTAAGAATAGTGATCTATATAGTACAAATGCTAGCTAAGTTAAGGCAGGGGTATAGGCCAGTGTTCTCTTCCCCACCCTCTTATTTCCAGTAGACTCATACCCAACAGGACCCGGCACTGCCAACCAGAGAAGGCAAAGAAGGAGACGGTGGAGAAGGCGGTGGATCCTATTATCGATCCATTGCATACGTTGTATCCATATCATAATATGTACATTTATATTGGCTCATGTCCAACATTACCGCCATGTTGACATTGATTATTGACTAGTTATTAATAGTAATCAATTACGGGGTCATTAGTTCATAGCCCATATATGGAGTTCCGCGTTACATAACTTACGGTAAATGGCCCGCCTGGCTGACCGCCCAACGACCCCCGCCCATTGACGTCAATAATGACGTATGTTCCCATAGTAACGCCAATAGGGACTTTCCATTGACGTCAATGGGTGGAGTATTTACGGTAAACTGCCCACTTGGCAGTACATCAAGTGTATCATATGCCAAGTACGCCCCCTATTGACGTCAATGACGGTAAATGGCCCGCCTGGCATTATGCCCAGTACATGACCTTATGGGACTTTCCTACTTGGCAGTACATCTACGTATTAGTCATCGCTATTACCATGGTGATGCGGTTTTGGCAGTACgTCAATGGGCGTGGATAGCGGTTTGACTCACGGGGATTTCCAAGTCTCCACCCCATTGACGTCAATGGGAGTTTGTTTTGGCACCAAAATCAACGGGACTTTCCAAAATGTCGTAACAACTCCGCCCCATTGACGCAAATGGGCGGTAGGCGTGTACGGTGGGAGGTCTATATAAGCAGAGCTCGTTTAGTGAACCGTCAGATCCAGCTGGCTaccggtaccatggctacctccgatgaagtgcggaaaaacctgatggatatgttccgcgaccgacaagcctttagtgagcatacatggaaaatgttgcttagcgtttgcaggtcatgggccgcttggtgcaaactgaacgggacccacaccctttatgcccctggtggttatgacattatggggtatctcgaccaaataggcaacaggcccaatccacaggttgaattggggcccgttgacacttcttgcgcccttatactctgtgacttgaaacaaaaggatacacccatagtgtacgccagcgaggcatttctctatatgacaggctactccaacgcagaggtcctgggtaggaactgtcggtttcttcaaagtccagatggtatggtcaaacccaagtccacaaggaaatacgtagactccaatacaatcaacacaatgaggaaggccatagatcgaaacgcagaggttcaggtggaagtggttaactttaagaaaaatggacaacgattcgttaacttcttgactatgatacctgtgcgggacgagactggggagtataggtattcaatggggttccagtgtgaaaccgaaggggggtcaggtggggtcccaaagaagaaacggaaggtcGGATCCGGCAGCGGCGCCACCAACTTCAGCCTGCTGAAGCAGGCCGGCGACGTGGAGGAGAACCCCGGCCCCCTCGAGGTGCCCAAGAAGAAGAGGAAAGTCGGCGGACATACTCTTTATGCCCCCGGTGGATATGACATTATGGGATATCTGAGGCAGATCAGGAACCGGCCAAACCCGCAGGTGGAACTGGGCCCCGTGGATACATCCTGCGCCTTGATTCTTTGTGACCTGAAACAGAAAGACACCCCGATAGTTTACGCGAGTGAAGCCTTCCTCTACATGACAGGTTACAGCAACGCAGAGGTGCTGGGCCGGAATTGCCGGTTTCTGCAAAGCCCTGACGGCATGGTGAAGCCCAAGAGCACCCGGAAGTACGTGGATAGTAACACAATCAATACTATGCGCAAGGCAATCGACAGGAATGCCGAGGTGCAGGTTGAAGTAGTCAATTTTAAAAAGAATGGACAGCGATTTGTTAATTTCCTGACTATGATACCTGTTAGGGACGAAACAGGCGAGTATCGATACTCTATGGGATTCCAGTGCGAAACAGAAGGTACCAACAGGAAATGGTTCCCTGCTGAACCTGAGGATGTGAGGGACTACCTCCTGTACCTGCAAGCCAGAGGCCTGGCTGTGAAGACCATCCAACAGCACCTGGGCCAGCTCAACATGCTGCACAGGAGATCTGGCCTGCCTCGCCCTTCTGACTCCAATGCTGTGTCCCTGGTGATGAGGAGAATCAGAAAGGAGAATGTGGATGCTGGGGAGAGAGCCAAGCAGGCCCTGGCCTTTGAACGCACTGACTTTGACCAAGTCAGATCCCTGATGGAGAACTCTGACAGATGCCAGGACATCAGGAACCTGGCCTTCCTGGGCATTGCCTACAACACCCTGCTGCGCATTGCCGAAATTGCCAGAATCAGAGTGAAGGACATCTCCCGCACCGATGGTGGGAGAATGCTGATCCACATTGGCAGGACCAAGACCCTGGTGTCCACAGCTGGTGTGGAGAAGGCCCTGTCCCTGGGGGTTACCAAGCTGGTGGAGAGATGGATCTCTGTGTCTGGTGTGGCTGATGACCCCAACAACTACCTGTTCTGCCGGGTCAGAAAGAATGGTGTGGCTGCCCCTTCTGCCACCTCCCAACTGTCCACCCGGGCCCTGGAAGGGATCTTTGAGGCCACCCACCGCCTGATCTATGGTGCCAAGGATGACTCTGGGCAGAGATACCTGGCCTGGTCTGGCCACTCTGCCAGAGTGGGTGCTGCCAGGGACATGGCCAGGGCTGGTGTGTCCATCCCTGAAATCATGCAGGCTGGTGGCTGGACCAATGTGAACATTGTGATGAACTACATCAGAAACCTGGACTCTGAGACTGGGGCCATGGTGAGGCTGCTCGAAGATGGGGACTAAGCTTGGATCCAATCAACCTCTGGATTACAAAATTTGTGAAAGATTGACTGGTATTCTTAACTATGTTGCTCCTTTTACGCTATGTGGATACGCTGCTTTAATGCCTTTGTATCATGCTATTGCTTCCCGTATGGCTTTCATTTTCTCCTCCTTGTATAAATCCTGGTTGCTGTCTCTTTATGAGGAGTTGTGGCCCGTTGTCAGGCAACGTGGCGTGGTGTGCACTGTGTTTGCTGACGCAACCCCCACTGGTTGGGGCATTGCCACCACCTGTCAGCTCCTTTCCGGGACTTTCGCTTTCCCCCTCCCTATTGCCACGGCGGAACTCATCGCCGCCTGCCTTGCCCGCTGCTGGACAGGGGCTCGGCTGTTGGGCACTGACAATTCCGTGGTGTTGTCGGGGAAGCTGACGTCCTTTCCATGGCTGCTCGCCTGTGTTGCCACCTGGATTCTGCGCGGGACGTCCTTCTGCTACGTCCCTTCGGCCCTCAATCCAGCGGACCTTCCTTCCCGCGGCCTGCTGCCGGCTCTGCGGCCTCTTCCGCGTCTTCGCCTTCGCCCTCAGACGAGTCGGATCTCCCTTTGGGCCGCCTCCCCGCCTGtttCGCCTCGCGACTCGAGCAAaCTcACaCTcAAGTTtATtTGCAAaCTcACaCTcAAGTTtATtTGCAAaCTcACaCTcAAGTCATGCATGgTCGAGTTTTATAAAAGAAAAGGGGGGACTGGAAGGGATTTATTACAGTGCAAGAAGACATAGAATCTTAGACATGTACTTAGAAAAGGAAGAAGGCATCATACCAGATTGGCAGGATTACACCTCAGGACCAGGAATTAGATACCCAAAGACATTTGGCTGGCTATGGAAATTAGTCCCTGTAAATGTATCAGATGAGGCACAGGAGGATGAGAGGCATTATTTAATGCAGCCAGCTCAAACTTCCAAGTGGGATGACCCTTGGGGAGAGGTTCTAGCGTGGAAGTTTGATCCAACTCTAGCCTACACTTATGAGGCATATGCTAGATACCCAGAAGAGTTGGAAGCAAGTCAGGCCTGTCAGAACTGCATTTCGCTCTGTATTCAGTCGCTCTGCGGAGAGGCTGGCAGATTGAGCCCTGGGAGGTTCTCTCCAGCACTAGCAGGTAGAGCCTGGGTGTTCCCTGCTAGACTCTCACCAGCACTTGGCCAGTGCTGGGCAGAGTGGCTCCACGCTTGCTTGCTTAAAGACCTCTTCAATAAAGCTGCCATTTTAGAAGTAAGCCAGTGTGTGTTCCCATCTCTCCTAGTCGCCGCCTGGTCAACTCGGTACTCGGTAATAAGAAGACCCTGGTCTGTTAGGACCCTTTCTGCTTTGAGAAACCGAAGCAGGAAAATCCCTAGCATGGTACCCAGCTTTTGTTCCCTTTAGTGAGGGTTAATTCCGAGCTTGGCGTAATCATGGTCATAGCTGTTTCCTGTGTGAAATTGTTATCCGCTCACAATTCCACACAACATACGAGCCGGAAGCATAAAGTGTAAAGCCTGGGGTGCCTAATGAGTGAGCTAACTCACATTAATTGCGTTGCGCTCACTGCCCGCTTTCCAGTCGGGAAACCTGTCGTGCCAGCTGCATTAATGAATCGGCCAACGCGCGGGGAGAGGCGGTTTGCGTATTGGGCGCTCTTCCGCTTCCTCGCTCACTGACTCGCTGCGCTCGGTCGTTCGGCTGCGGCGAGCGGTATCAGCTCACTCAAAGGCGGTAATACGGTTATCCACAGAATCAGGGGATAACGCAGGAAAGAACATGTGAGCAAAAGGCCAGCAAAAGGCCAGGAACCGTAAAAAGGCCGCGTTGCTGGCGTTTTTCCATAGGCTCCGCCCCCCTGACGAGCATCACAAAAATCGACGCTCAAGTCAGAGGTGGCGAAACCCGACAGGACTATAAAGATACCAGGCGTTTCCCCCTGGAAGCTCCCTCGTGCGCTCTCCTGTTCCGACCCTGCCGCTTACCGGATACCTGTCCGCCTTTCTCCCTTCGGGAAGCGTGGCGCTTTCTCATAGCTCACGCTGTAGGTATCTCAGTTCGGTGTAGGTCGTTCGCTCCAAGCTGGGCTGTGTGCACGAACCCCCCGTTCAGCCCGACCGCTGCGCCTTATCCGGTAACTATCGTCTTGAGTCCAACCCGGTAAGACACGACTTATCGCCACTGGCAGCAGCCACTGGTAACAGGATTAGCAGAGCGAGGTATGTAGGCGGTGCTACAGAGTTCTTGAAGTGGTGGCCTAACTACGGCTACACTAGAAGGACAGTATTTGGTATCTGCGCTCTGCTGAAGCCAGTTACCTTCGGAAAAAGAGTTGGTAGCTCTTGATCCGGCAAACAAACCACCGCTGGTAGCGGTGGTTTTTTTGTTTGCAAGCAGCAGATTACGCGCAGAAAAAAAGGATCTCAAGAAGATCCTTTGATCTTTTCTACGGGGTCTGACGCTCAGTGGAACGAAAACTCACGTTAAGGGATTTTGGTCATGAGATTATCAAAAAGGATCTTCACCTAGATCCTTTTAAATTAAAAATGAAGTTTTAAATCAATCTAAAGTATATATGAGTAAACTTGGTCTGACAGTTACCAATGCTTAATCAGTGAGGCACCTATCTCAGCGATCTGTCTATTTCGTTCATCCATAGTTGCCTGACTCCCCGTCGTGTAGATAACTACGATACGGGAGGGCTTACCATCTGGCCCCAGTGCTGCAATGATACCGCGAGACCCACGCTCACCGGCTCCAGATTTATCAGCAATAAACCAGCCAGCCGGAAGGGCCGAGCGCAGAAGTGGTCCTGCAACTTTATCCGCCTCCATCCAGTCTATTAATTGTTGCCGGGAAGCTAGAGTAAGTAGTTCGCCAGTTAATAGTTTGCGCAACGTTGTTGCCATTGCTACAGGCATCGTGGTGTCACGCTCGTCGTTTGGTATGGCTTCATTCAGCTCCGGTTCCCAACGATCAAGGCGAGTTACATGATCCCCCATGTTGTGCAAAAAAGCGGTTAGCTCCTTCGGTCCTCCGATCGTTGTCAGAAGTAAGTTGGCCGCAGTGTTATCACTCATGGTTATGGCAGCACTGCATAATTCTCTTACTGTCATGCCATCCGTAAGATGCTTTTCTGTGACTGGTGAGTACTCAACCAAGTCATTCTGAGAATAGTGTATGCGGCGACCGAGTTGCTCTTGCCCGGCGTCAATACGGGATAATACCGCGCCACATAGCAGAACTTTAAAAGTGCTCATCATTGGAAAACGTTCTTCGGGGCGAAAACTCTCAAGGATCTTACCGCTGTTGAGATCCAGTTCGATGTAACCCACTCGTGCACCCAACTGATCTTCAGCATCTTTTACTTTCACCAGCGTTTCTGGGTGAGCAAAAACAGGAAGGCAAAATGCCGCAAAAAAGGGAATAAGGGCGACACGGAAATGTTGAATACTCATACTCTTCCTTTTTCAATATTATTGAAGCATTTATCAGGGTTATTGTCTCATGAGCGGATACATATTTGAATGTATTTAGAAAAATAAACAAATAGGGGTTCCGCGCACATTTCCCCGAAAAGTGCCACCTG

Supplementary Text S2: Peptide sequences. Blue, AsLOV2 residues (from iLID sequence PDB 4WF0); red, single-site specific residues; green, VVD residues; yellow, Flag epitope tag; grey, flexible linker.

>CreWT

  1 MSNLLTVHQNLPALPVDATS

 21 DEVRKNLMDMFRDRQAFSEH

 41 TWKMLLSVCRSWAAWCKLNN

 61 RKWFPAEPEDVRDYLLYLQA

 81 RGLAVKTIQQHLGQLNMLHR

101 RSGLPRPSDSNAVSLVMRRI

121 RKENVDAGERAKQALAFERT

141 DFDQVRSLMENSDRCQDIRN

161 LAFLGIAYNTLLRIAEIARI

181 RVKDISRTDGGRMLIHIGRT

201 KTLVSTAGVEKALSLGVTKL

221 VERWISVSGVADDPNNYLFC

241 RVRKNGVAAPSATSQLSTRA

261 LEGIFEATHRLIYGAKDDSG

281 QRYLAWSGHSARVGAARDMA

301 RAGVSIPEIMQAGGWTNVNI

321 VMNYIRNLDSETGAMVRLLE

341 DGD

>LiCre (from pGY466)

MRGSHHHHHHGSLATTLERIEKNFVITDPRLPDNPIIFASDSFLQLTEYSREEILGRNCRFLQGPETDRATVRKIRDAIDNQTEVTVQLINYTKSGKKFWNVFHLQPMRDYKGDVQYFIGVQLDGTERLHGAAEREAVCLIKKTAFQIDSDEVRKNLMDMFRDRQAFSEHTWKMLLSVCRSWAAWCKLNNRKWFPAEPEDVRDYLLYLQARGLAVKTIQQHLGQLNMLHRRSGLPRPSDSNAVSLVMRRIRKENVDAGERAKQALAFERTDFDQVRSLMENSDRCQDIRNLAFLGIAYNTLLRIAEIARIRVKDISRTDGGRMLIHIGRTKTLVSTAGVEKALSLGVTKLVERWISVSGVADDPNNYLFCRVRKNGVAAPSATSQLSTRALEGIFEATHRLIYGAKDDSGQRYLAWSGHSARVGAARDMARAGVSIPEIMQAGGWTNVNIVMNYIRNLDSETGAMVRLLAAGD

>LOV_Cre17

MRGSHHHHHHGSLATTLERIEKNFVITDPRLPDNPIIFASDSFLQLTEYSREEILGRNCRFLQGPETDRATVRKIRDAIDNQTEVTVQLINYTKSGKKFWNVFHLQPMRDYKGDVQYFIGVQLDGTERLHGAAEREAVCLIKKTAFQIADATSDEVRKNLMDMFRDRQAFSEHTWKMLLSVCRSWAAWCKLNNRKWFPAEPEDVRDYLLYLQARGLAVKTIQQHLGQLNMLHRRSGLPRPSDSNAVSLVMRRIRKENVDAGERAKQALAFERTDFDQVRSLMENSDRCQDIRNLAFLGIAYNTLLRIAEIARIRVKDISRTDGGRMLIHIGRTKTLVSTAGVEKALSLGVTKLVERWISVSGVADDPNNYLFCRVRKNGVAAPSATSQLSTRALEGIFEATHRLIYGAKDDSGQRYLAWSGHSARVGAARDMARAGVSIPEIMQAGGWTNVNIVMNYIRNLDSETGAMVRLLEDGD

>LOV_Cre19

MRGSHHHHHHGSLATTLERIEKNFVITDPRLPDNPIIFASDSFLQLTEYSREEILGRNCRFLQGPETDRATVRKIRDAIDNQTEVTVQLINYTKSGKKFWNVFHLQPMRDYKGDVQYFIGVQLDGTERLHGAAEREAVCLIKKTAFQIATSDEVRKNLMDMFRDRQAFSEHTWKMLLSVCRSWAAWCKLNNRKWFPAEPEDVRDYLLYLQARGLAVKTIQQHLGQLNMLHRRSGLPRPSDSNAVSLVMRRIRKENVDAGERAKQALAFERTDFDQVRSLMENSDRCQDIRNLAFLGIAYNTLLRIAEIARIRVKDISRTDGGRMLIHIGRTKTLVSTAGVEKALSLGVTKLVERWISVSGVADDPNNYLFCRVRKNGVAAPSATSQLSTRALEGIFEATHRLIYGAKDDSGQRYLAWSGHSARVGAARDMARAGVSIPEIMQAGGWTNVNIVMNYIRNLDSETGAMVRLLEDGD

>LOV_Cre27

MRGSHHHHHHGSLATTLERIEKNFVITDPRLPDNPIIFASDSFLQLTEYSREEILGRNCRFLQGPETDRATVRKIRDAIDNQTEVTVQLINYTKSGKKFWNVFHLQPMRDYKGDVQYFIGVQLDGTERLHGAAEREAVCLIKKTAFQIALMDMFRDRQAFSEHTWKMLLSVCRSWAAWCKLNNRKWFPAEPEDVRDYLLYLQARGLAVKTIQQHLGQLNMLHRRSGLPRPSDSNAVSLVMRRIRKENVDAGERAKQALAFERTDFDQVRSLMENSDRCQDIRNLAFLGIAYNTLLRIAEIARIRVKDISRTDGGRMLIHIGRTKTLVSTAGVEKALSLGVTKLVERWISVSGVADDPNNYLFCRVRKNGVAAPSATSQLSTRALEGIFEATHRLIYGAKDDSGQRYLAWSGHSARVGAARDMARAGVSIPEIMQAGGWTNVNIVMNYIRNLDSETGAMVRLLEDGD

>LOV_Cre32 (from pGY415)

MRGSHHHHHHGSLATTLERIEKNFVITDPRLPDNPIIFASDSFLQLTEYSREEILGRNCRFLQGPETDRATVRKIRDAIDNQTEVTVQLINYTKSGKKFWNVFHLQPMRDYKGDVQYFIGVQLDGTERLHGAAEREAVCLIKKTAFQIARDRQAFSEHTWKMLLSVCRSWAAWCKLNNRKWFPAEPEDVRDYLLYLQARGLAVKTIQQHLGQLNMLHRRSGLPRPSDSNAVSLVMRRIRKENVDAGERAKQALAFERTDFDQVRSLMENSDRCQDIRNLAFLGIAYNTLLRIAEIARIRVKDISRTDGGRMLIHIGRTKTLVSTAGVEKALSLGVTKLVERWISVSGVADDPNNYLFCRVRKNGVAAPSATSQLSTRALEGIFEATHRLIYGAKDDSGQRYLAWSGHSARVGAARDMARAGVSIPEIMQAGGWTNVNIVMNYIRNLDSETGAMVRLLEDGD

>LOV_CreAA21 (Fig. 2c, construct j)

MRGSHHHHHHGSLATTLERIEKNFVITDPRLPDNPIIFASDSFLQLTEYSREEILGRNCRFLQGPETDRATVRKIRDAIDNQTEVTVQLINYTKSGKKFWNVFHLQPMRDYKGDVQYFIGVQLDGTERLHGAAEREAVCLIKKTAFQIADEVRKNLMDMFRDRQAFSEHTWKMLLSVCRSWAAWCKLNNRKWFPAEPEDVRDYLLYLQARGLAVKTIQQHLGQLNMLHRRSGLPRPSDSNAVSLVMRRIRKENVDAGERAKQALAFERTDFDQVRSLMENSDRCQDIRNLAFLGIAYNTLLRIAEIARIRVKDISRTDGGRMLIHIGRTKTLVSTAGVEKALSLGVTKLVERWISVSGVADDPNNYLFCRVRKNGVAAPSATSQLSTRALEGIFEATHRLIYGAKDDSGQRYLAWSGHSARVGAARDMARAGVSIPEIMQAGGWTNVNIVMNYIRNLDSETGAMVRLLAAGD

>LOV_CreAA22 (Fig. 2c, construct k)

MRGSHHHHHHGSLATTLERIEKNFVITDPRLPDNPIIFASDSFLQLTEYSREEILGRNCRFLQGPETDRATVRKIRDAIDNQTEVTVQLINYTKSGKKFWNVFHLQPMRDYKGDVQYFIGVQLDGTERLHGAAEREAVCLIKKTAFQIAEVRKNLMDMFRDRQAFSEHTWKMLLSVCRSWAAWCKLNNRKWFPAEPEDVRDYLLYLQARGLAVKTIQQHLGQLNMLHRRSGLPRPSDSNAVSLVMRRIRKENVDAGERAKQALAFERTDFDQVRSLMENSDRCQDIRNLAFLGIAYNTLLRIAEIARIRVKDISRTDGGRMLIHIGRTKTLVSTAGVEKALSLGVTKLVERWISVSGVADDPNNYLFCRVRKNGVAAPSATSQLSTRALEGIFEATHRLIYGAKDDSGQRYLAWSGHSARVGAARDMARAGVSIPEIMQAGGWTNVNIVMNYIRNLDSETGAMVRLLAAGD

> NLS-pMag-CreC60-343 (from pGY491)

MVPKKKRKVGGHTLYAPGGYDIMGYLRQIRNRPNPQVELGPVDTSCALILCDLKQKDTPIVYASEAFLYMTGYSNAEVLGRNCRFLQSPDGMVKPKSTRKYVDSNTINTMRKAIDRNAEVQVEVVNFKKNGQRFVNFLTMIPVRDETGEYRYSMGFQCETEGTNRKWFPAEPEDVRDYLLYLQARGLAVKTIQQHLGQLNMLHRRSGLPRPSDSNAVSLVMRRIRKENVDAGERAKQALAFERTDFDQVRSLMENSDRCQDIRNLAFLGIAYNTLLRIAEIARIRVKDISRTDGGRMLIHIGRTKTLVSTAGVEKALSLGVTKLVERWISVSGVADDPNNYLFCRVRKNGVAAPSATSQLSTRALEGIFEATHRLIYGAKDDSGQRYLAWSGHSARVGAARDMARAGVSIPEIMQAGGWTNVNIVMNYIRNLDSETGAMVRLLEDGD

> CreN18-59-nMag-NLS (from pGY501)

MATSDEVRKNLMDMFRDRQAFSEHTWKMLLSVCRSWAAWCKLNGTHTLYAPGGYDIMGYLDQIGNRPNPQVELGPVDTSCALILCDLKQKDTPIVYASEAFLYMTGYSNAEVLGRNCRFLQSPDGMVKPKSTRKYVDSNTINTMRKAIDRNAEVQVEVVNFKKNGQRFVNFLTMIPVRDETGEYRYSMGFQCETEGGSGGVPKKKRKV

> CRY2L348F-CreN19-104 (from pGY531)

MKMDKKTIVWFRRDLRIEDNPALAAAAHEGSVFPVFIWCPEEEGQFYPGRASRWWMKQSLAHLSQSLKALGSDLTLIKTHNTISAILDCIRVTGATKVVFNHLYDPVSLVRDHTVKEKLVERGISVQSYNGDLLYEPWEIYCEKGKPFTSFNSYWKKCLDMSIESVMLPPPWRLMPITAAAEAIWACSIEELGLENEAEKPSNALLTRAWSPGWSNADKLLNEFIEKQLIDYAKNSKKVVGNSTSLLSPYLHFGEISVRHVFQCARMKQIIWARDKNSEGEESADLFLRGIGLREYSRYICFNFPFTHEQSLLSHLRFFPWDADVDKFKAWRQGRTGYPLVDAGMREFWATGWMHNRIRVIVSSFAVKFLLLPWKWGMKYFWDTLLDADLECDILGWQYISGSIPDGHELDRLDNPALQGAKYDPEGEYIRQWLPELARLPTEWIHHPWDAPLTVLKASGVELGTNYAKPIVDIDTARELLAKAISRTREAQIMIGAAPDEIVADSFEALGANTIKEPGLCPSVSSNDQQVPSAVRYNGSKRVKPEEEEERDMKKSRGFDERELFSTAESSSSSSVFFVSQSCSLASEGKNLEGIQDSSDQITTSLGKNGCKGGGGSGGGGSGGGGRTSDEVRKNLMDMFRDRQAFSEHTWKMLLSVCRSWAAWCKLNNRKWFPAEPEDVRDYLLYLQARGLAVKTIQQHLGQLNMLHRRSGL

> CIB1-CreC106-343 (from pGY532)

MNGAIGGDLLLNFPDMSVLERQRAHLKYLNPTFDSPLAGFFADSSMITGGEMDSYLSTAGLNLPMMYGETTVEGDSRLSISPETTLGTGNFKKRKFDTETKDCNEKKKKMTMNRDDLVEEGEEEKSKITEQNNGSTKSIKKMKHKAKKEENNFSNDSSKVTKELEKTDYIHVRARRGQATDSHSIAERVRREKISERMKFLQDLVPGCDKITGKAGMLDEIINYVQSLQRQIEFLSMKLAIVNPRPDFDMDDIFAKEVASTPMTVVPSPEMVLSGYSHEMVHSGYSSEMVNSGYLHVNPMQQVNTSSDPLSCFNNGEAPSMWDSHVQNLYGNLGVGGGGSGGGGSGGGGRRPSDSNAVSLVMRRIRKENVDAGERAKQALAFERTDFDQVRSLMENSDRCQDIRNLAFLGIAYNTLLRIAEIARIRVKDISRTDGGRMLIHIGRTKTLVSTAGVEKALSLGVTKLVERWISVSGVADDPNNYLFCRVRKNGVAAPSATSQLSTRALEGIFEATHRLIYGAKDDSGQRYLAWSGHSARVGAARDMARAGVSIPEIMQAGGWTNVNIVMNYIRNLDSETGAMVRLLEDGD

>Cre-4-VVD

MSNLLTVHQNLPALPVDATSDEVRKNLMDMFRDRQAFSEHTWKMLLSVCRSWAAWCKLNN

RKWFPAEPEDVRDYLLYLQARGLAVKTIQQHLGQLNMLHRRSGLPRPSDSNAVSLVMRRI

RKENVDAGERAKQALAFERTDFDQVRSLMENSDRCQDIRNLAFLGIAYNTLLRIAEIARI

RVKDISRTDGGRMLIHIGRTKTLVSTAGVEKALSLGVTKLVERWISVSGVADDPNNYLFC

RVRKNGVAAPSATSQLSTRALEGIFEATHRLIYGAKDDSGQRYLAWSGHSARVGAARDMA

RAGVSIPEIMQAGGWTNVNIVMNYIRNLDSETGAMVRLLEDGDGGSGSHTVNSSTMNPWE

VEAYQQYHYDPRTAPTANPLFFHTLYAPGGYDIMGYLIQIMNRPNPQVELGPVDTSCALI

LCDLKQKDTPIVYASEAFLYMTGYSNAEVLGRNCRFLQSPDGMVKPKSTRKYVDSNTINT

IRKAIDRNAEVQVEVVNFKKNGQRFVNFLTIIPVRDETGEYRYSMGFQCETEDYKDDDDK

>CreAA-0-VVD

MSNLLTVHQNLPALPVDATSDEVRKNLMDMFRDRQAFSEHTWKMLLSVCRSWAAWCKLNN

RKWFPAEPEDVRDYLLYLQARGLAVKTIQQHLGQLNMLHRRSGLPRPSDSNAVSLVMRRI

RKENVDAGERAKQALAFERTDFDQVRSLMENSDRCQDIRNLAFLGIAYNTLLRIAEIARI

RVKDISRTDGGRMLIHIGRTKTLVSTAGVEKALSLGVTKLVERWISVSGVADDPNNYLFC

RVRKNGVAAPSATSQLSTRALEGIFEATHRLIYGAKDDSGQRYLAWSGHSARVGAARDMA

RAGVSIPEIMQAGGWTNVNIVMNYIRNLDSETGAMVRLLEDGDSHTVNSSTMNPWE

VEAYQQYHYDPRTAPTANPLFFHTLYAPGGYDIMGYLIQIMNRPNPQVELGPVDTSCALI

LCDLKQKDTPIVYASEAFLYMTGYSNAEVLGRNCRFLQSPDGMVKPKSTRKYVDSNTINT

IRKAIDRNAEVQVEVVNFKKNGQRFVNFLTIIPVRDETGEYRYSMGFQCETEDYKDDDDK

>CreAA-2-VVD

MSNLLTVHQNLPALPVDATSDEVRKNLMDMFRDRQAFSEHTWKMLLSVCRSWAAWCKLNN

RKWFPAEPEDVRDYLLYLQARGLAVKTIQQHLGQLNMLHRRSGLPRPSDSNAVSLVMRRI

RKENVDAGERAKQALAFERTDFDQVRSLMENSDRCQDIRNLAFLGIAYNTLLRIAEIARI

RVKDISRTDGGRMLIHIGRTKTLVSTAGVEKALSLGVTKLVERWISVSGVADDPNNYLFC

RVRKNGVAAPSATSQLSTRALEGIFEATHRLIYGAKDDSGQRYLAWSGHSARVGAARDMA

RAGVSIPEIMQAGGWTNVNIVMNYIRNLDSETGAMVRLLEDGDGGSHTVNSSTMNPWE

VEAYQQYHYDPRTAPTANPLFFHTLYAPGGYDIMGYLIQIMNRPNPQVELGPVDTSCALI

LCDLKQKDTPIVYASEAFLYMTGYSNAEVLGRNCRFLQSPDGMVKPKSTRKYVDSNTINT

IRKAIDRNAEVQVEVVNFKKNGQRFVNFLTIIPVRDETGEYRYSMGFQCETEDYKDDDDK

>CreXX-4-VVD (where XX was one of AA, AD, AR, EA, RA, RR)

MSNLLTVHQNLPALPVDATSDEVRKNLMDMFRDRQAFSEHTWKMLLSVCRSWAAWCKLNN

RKWFPAEPEDVRDYLLYLQARGLAVKTIQQHLGQLNMLHRRSGLPRPSDSNAVSLVMRRI

RKENVDAGERAKQALAFERTDFDQVRSLMENSDRCQDIRNLAFLGIAYNTLLRIAEIARI

RVKDISRTDGGRMLIHIGRTKTLVSTAGVEKALSLGVTKLVERWISVSGVADDPNNYLFC

RVRKNGVAAPSATSQLSTRALEGIFEATHRLIYGAKDDSGQRYLAWSGHSARVGAARDMA

RAGVSIPEIMQAGGWTNVNIVMNYIRNLDSETGAMVRLLXXGDGGSGSHTVNSSTMNPWE

VEAYQQYHYDPRTAPTANPLFFHTLYAPGGYDIMGYLIQIMNRPNPQVELGPVDTSCALI

LCDLKQKDTPIVYASEAFLYMTGYSNAEVLGRNCRFLQSPDGMVKPKSTRKYVDSNTINT

IRKAIDRNAEVQVEVVNFKKNGQRFVNFLTIIPVRDETGEYRYSMGFQCETEDYKDDDDK
